# Multifunctional Metal-Coordinated Hydrogels: Controlled Synthesis and Advances in Anti-Inflammatory, Anticancer, and Antibacterial Applications

**DOI:** 10.1021/acsomega.6c06631

**Published:** 2026-09-08

**Authors:** Junjia Gao, Zeng-En Jin, Yikuan Li, Chenxin Gu, Chunmei Zhi, Wei Wu, Qing Chen, Sen Gao

**Affiliations:** † School of Pharmacy, 70577 Shenyang Medical College, Shenyang 110034, People’s Republic of China; ‡ The Second Affiliated Hospital of Shenyang Medical College, Shenyang 110024, People’s Republic of China; § Central Hospital Affiliated to Shenyang Medical College, Shenyang 110024, People’s Republic of China; ∥ Research Center for Analytical Sciences, Department of Chemistry, College of Sciences, 12434 Northeastern University, Shenyang 110819, People’s Republic of China

## Abstract

Hydrogels possess exceptional biocompatibility and have therefore been widely utilized in biomedical applications. However, their broader development is often constrained by limited intrinsic functionality and inadequate dynamic responsiveness. Metal complexes offer a promising solution to these limitations by introducing dynamic coordination interactions, catalytic properties, and multi-stimuli responsiveness, enabling new avenues for hydrogel functionalization. This review begins with a systematic examination of four principal strategies for integrating metal complexes into hydrogel matrices: physical embedding, chemical grafting, coordination-based cross-linking, and *in situ* complex formation. This review methodically compares these four integration strategies rather than treating them as interchangeable routes. Emerging evidence indicates that incorporating metal complexes can endow hydrogels with enhanced biomedical capabilities, including anti-inflammatory, anticancer, and antibacterial activities. Despite these advances, several challenges persist, particularly with respect to biocompatibility, long-term structural and functional stability, and scalable manufacturing. Consequently, we propose the implementation of application-specific design criteria and a translational framework covering metal pharmacokinetics, biodegradation, manufacturing reproducibility, and long-term safety. Future research is therefore expected to focus on the development of biomimetic, multi-responsive hydrogel systems that support precision therapeutics and regenerative medicine. This analysis distinguishes metal-coordinated hydrogels from metal-free hydrogels and unconfined metal nanomaterials, while identifying the evidence still required for clinical development.

## Graphical abstract

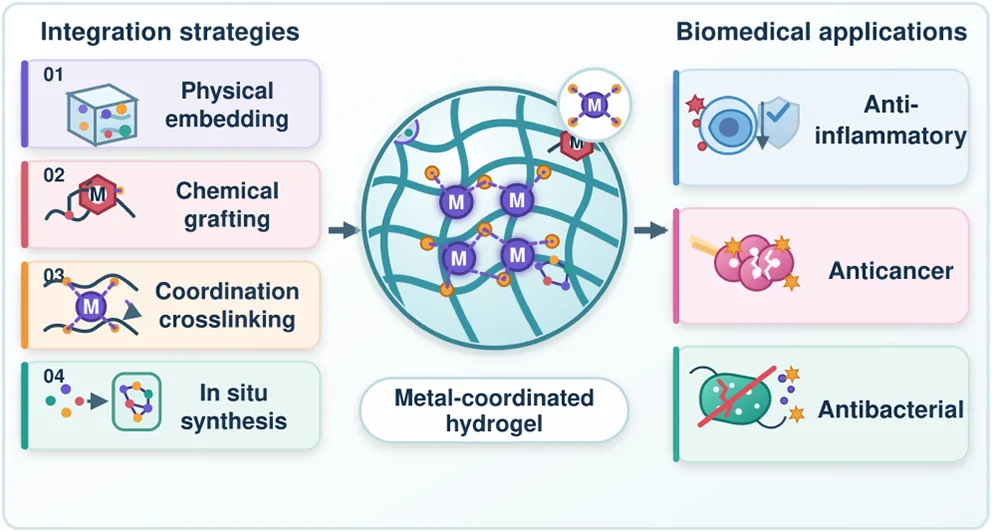

## 1 Introduction

### 1.1 Hydrogels

Hydrogels are hydrated three-dimensional polymer networks whose behavior is determined not only by water content, but also by polymer origin, network topology, and crosslink dynamics.^1–5^ Their favorable characteristics, including adjustable mechanical properties,^2,6–8^ excellent biocompatibility,^9^ and tunable biodegradability,^10^ make them highly promising materials for a wide range of biomedical applications,^11–13^ particularly in drug delivery systems^14,15^ and antibacterial applications.^9^

As biocompatible soft materials, the hydrophilic nature of hydrogels provides an ideal microenvironment for cellular growth. It is important to note that biocompatibility is not an intrinsic property of the term hydrogel. Instead, it is dependent on the purity of the polymer, the presence of residual initiators or crosslinkers, osmotic pressure, stiffness, degradation products, and residence time. Moreover, their three-dimensional network structure closely resembles the natural extracellular matrix (ECM), supporting key cellular processes such as adhesion, proliferation, and differentiation.^16–27^ The mechanical properties and microarchitecture of hydrogels can be precisely engineered through modulation of cross-linking density, polymer composition, and incorporated functional groups, to meet specific biological requirements. When employed as drug-delivery platforms, hydrogels facilitate localized and sustained release of therapeutic agents, which not only minimizes systemic toxicity but also improves therapeutic outcomes, particularly in cancer treatment and chronic wound management.^28–31^ Furthermore, functionalized hydrogels incorporating antimicrobial agents and antioxidant moieties provide dual functionality in wound dressings by simultaneously preventing infection and reducing oxidative stress, addressing multiple therapeutic needs at once.^32,33^ Nevertheless, network design often involves coupled trade-offs. For instance, increasing crosslink density may improve shape retention and suppress cargo leakage, concurrently reducing cell infiltration and solute diffusion. Highly dynamic networks can be injectable and self-healing, yet creep or erode under sustained load.^5,6,8^

Despite their considerable promise in biomedical applications, hydrogels face several key obstacles that hinder successful clinical translation. Translation is consequently limited by more than inadequate mechanical strength: batch-to-batch variation in natural polymers, incomplete reporting of gelation and rheology, sterilization-induced changes, poorly resolved degradation pathways, and the absence of clinically relevant comparators collectively complicate interpretation.^5,34,35^ A major limitation is their insufficient mechanical strength, particularly in systems derived from natural polymers.^36–38^ Many hydrogels exhibit inherently monofunctional behavior. While they may effectively function as drug carriers or structural matrices, they often fall short in addressing multiple concurrent clinical requirements, such as providing antibacterial and antioxidant activity while simultaneously promoting vascularization.^2^ Infection risk poses another significant challenge. Owing to their high water content and intrinsic biocompatibility, hydrogels can inadvertently support bacterial adhesion and biofilm formation, which may provoke severe inflammatory responses in chronic wounds or on implanted devices.^20,32,39^ Furthermore, the lack of intelligent or adaptive responsiveness limits their performance within dynamic physiological environments. Conventional hydrogels typically do not exhibit adequate sensitivity to stimuli such as pH, temperature, or biological signals, restricting their ability to facilitate on-demand drug release or modulate mechanical properties in real time.^40^ These limitations highlight the pressing need for the development of next-generation hydrogels that are multifunctional, mechanically robust, and capable of responding to complex biological cues.

### 1.2 Metal Complexes

Metal complexes are formed through the self-assembly of central metal ions with organic or inorganic ligands, including polyphenols, phenanthroline, and Schiff bases. The structural dimensionality of these complexes is adjustable, ranging from nanoclusters to 3D metal-organic frameworks (MOFs), conferring distinctive physicochemical characteristics to these materials.^41–43^ In the field of catalysis, dynamic coordination structural evolution has been observed during the redox reactions of copper complexes.^44^ In optical research, copper nanoclusters exhibit tunable luminescence, which is attributed to quantum size effects.^45^ These structural and functional variabilities highlight their broad applicability across multiple areas, including catalysis, optics, magnetism, and biomedicine, particularly in cancer theranostics, antimicrobial coatings, and antioxidant therapies.^46–49^

The relationship between the structure and function of metal complexes is fundamental to their biomedical applications. The catalytic behavior and biological activities of these complexes are largely governed by the electronic configurations, redox potentials, and coordination properties of their constituent metal ions. For instance, Ag^+^ and Cu^2+^ exhibit broad-spectrum antimicrobial activity, primarily due to their capacity to disrupt cell membrane integrity and inhibit key enzymes in the respiratory chain.^46,49–54^ Similarly, Ce^3+^ and Mn^2+^ can improve antioxidant capacity, offering promising strategies for disease prevention and treatment.^54–57^ Dynamic coordination bonds impart self-healing behavior and stimulus responsiveness to these materials, facilitating the design of smart drug-delivery systems.^41,51,58,59^ Metal ions can also be integrated with antibiotics to help overcome drug resistance, as exemplified by silver–ampicillin complexes.^49^ Similarly, ligand diversity plays a crucial role in the biomedical performance of metal complexes. For instance, tannic acid effectively scavenges reactive oxygen species (ROS) through hydrogen atom transfer from its phenolic hydroxyl groups.^41,60,61^ However, the clinical application of metal complexes is limited by several challenges, including uncontrolled biodistribution, unclear long-term toxicity profiles, and insufficiently characterized metabolic pathways.^38,45,62–65^ Future research should therefore focus on the rational design and synthesis of ligands and complexes, a deeper elucidation of structure–function relationships, and comprehensive biosafety assessments to facilitate the clinical translation of safe and effective metal-based therapeutics.

### 1.3 Mechanisms of Synergy

Metal coordination can enter a hydrogel in two fundamentally different roles. A preformed complex or metal-containing particle may act mainly as a functional cargo, with the polymer matrix controlling localization and transport.^4,5,31^ Alternatively, metal–ligand bonds may serve as network junctions and directly determine gelation, toughness, self-healing, and stimulus response.^18,64,66–68^ Some systems integrate both roles. Discerning between them is critical, because comparable elemental compositions can yield substantially different release profiles and biological exposures.

The therapeutic benefit derives from this coupling rather than from the metal alone. Confinement may maintain a high local concentration, limit systemic exposure, and enable externally triggered activity, while coordination can confer sacrificial bonds or redox- and pH-responsive junctions.^18,64,66–68^ The same interactions can also be detrimental: tight chelation may inhibit a metal-dependent catalytic or antimicrobial pathway, whereas weak binding may cause burst release and reduce the safety margin.^4,18,41,49^ Thus, loading content by itself is a poor predictor of efficacy. Compared with a metal-free hydrogel, the coordinated system can introduce catalytic, optical, magnetic, or ion-mediated biological functions without a separate device. Compared with free ions, isolated complexes, or unconfined nanoparticles, the hydrogel can enhance local retention and conformal contact.^3–5,8,41,69^ This advantage is context-dependent, not universal: covalent drug conjugates may offer more precise composition, and conventional dressings may be preferred when metal-mediated activity does not warrant the added toxicological and manufacturing complexity.^5,35,70^

### 1.4 Literature Organization

Recent reviews have examined smart injectable hydrogels, therapeutic hydrogels, metal-ion-containing scaffolds, or MOF–hydrogel composites as separate topics.^3–5,8,69^ What remains less clear is how the mode of metal integration affects both material performance and biological exposure across disease settings. We address this gap by applying one operational framework—physical embedding, chemical grafting, coordination crosslinking, and *in situ* formation—to molecular complexes, coordination polymers, and MOF-containing hydrogels. We then connect metal and ligand chemistry to mechanics, release, degradation, and therapeutic function; benchmark quantitative end points across anti-inflammatory, anticancer, and antibacterial studies; and evaluate manufacturing, sterilization, pharmacokinetics, and regulatory evidence (see Figure 1). The purpose is therefore not to compile another list of formulations, but to assess when metal coordination provides a defensible advantage and when it introduces an avoidable liability.

**Figure 1:**
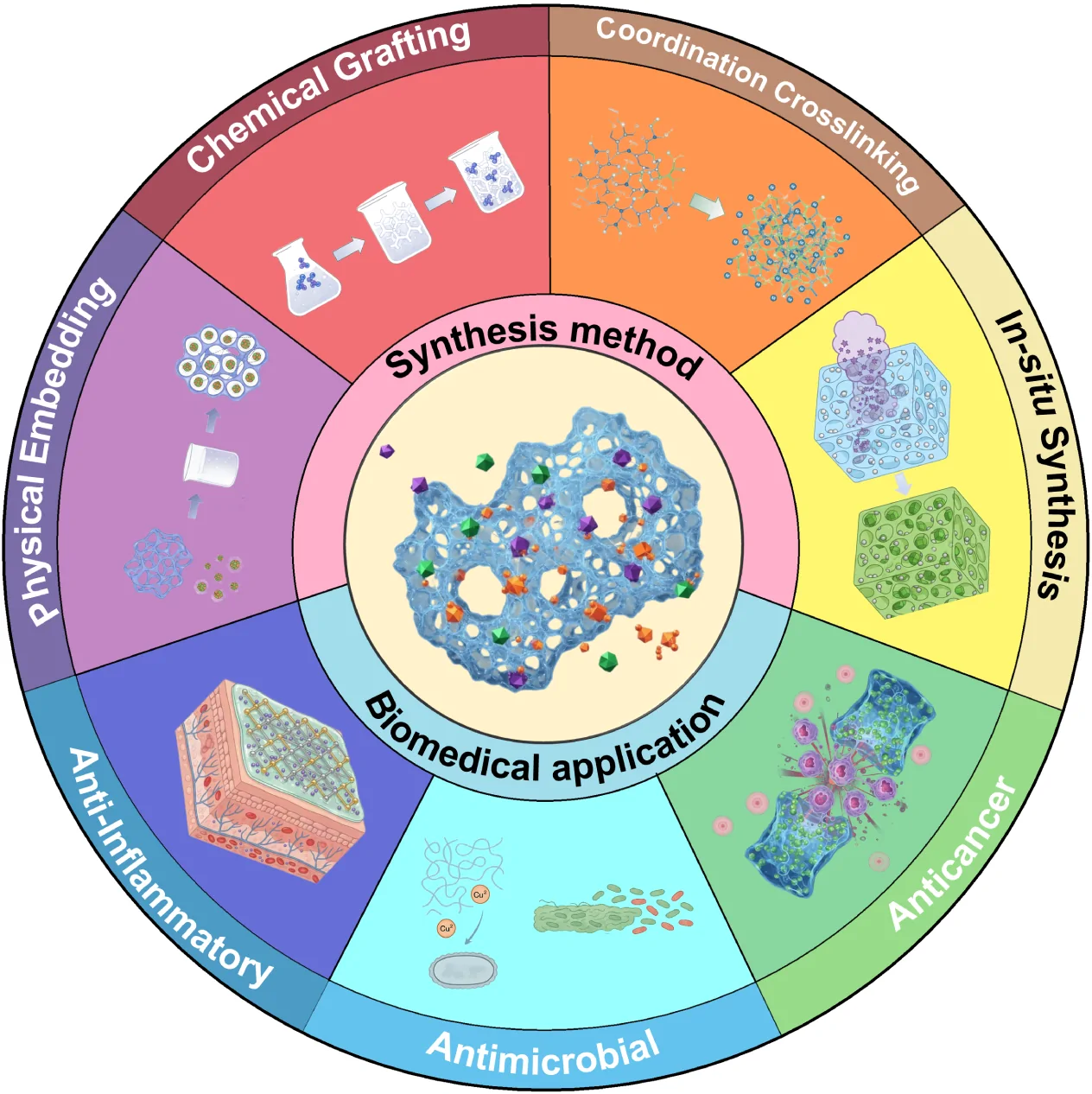
Schematic illustration of the synthesis strategy and biomedical applications of metal-coordinated hydrogels.

## 2 Design Strategies

### 2.1 Physical Embedding

#### 2.1.1 Principles

The physical embedding of metal complexes into hydrogels relies on non-covalent interactions, including van der Waals forces, hydrogen bonding, capillary effects, and steric confinement, to retain the complexes within the three-dimensional hydrogel network, as schematically illustrated in Figure 2. This method obviates the need for chemical modification, thus preserving the intrinsic structure and active sites of metal complexes, and is typically implemented through a three-step process. First, the metal complexes are synthesized, with metal ions coordinating with ligands to form stable architectures such as metal–organic frameworks (MOFs) or mono- and dinuclear complexes. Second, the complexes are dispersed into the hydrogel precursor solution, commonly via ultrasonication, followed by gelation, which can proceed through either physical or chemical cross-linking. Third, the complexes are physically entrapped within the porous hydrogel matrix and stabilized through weak, reversible interactions.

**Figure 2:**
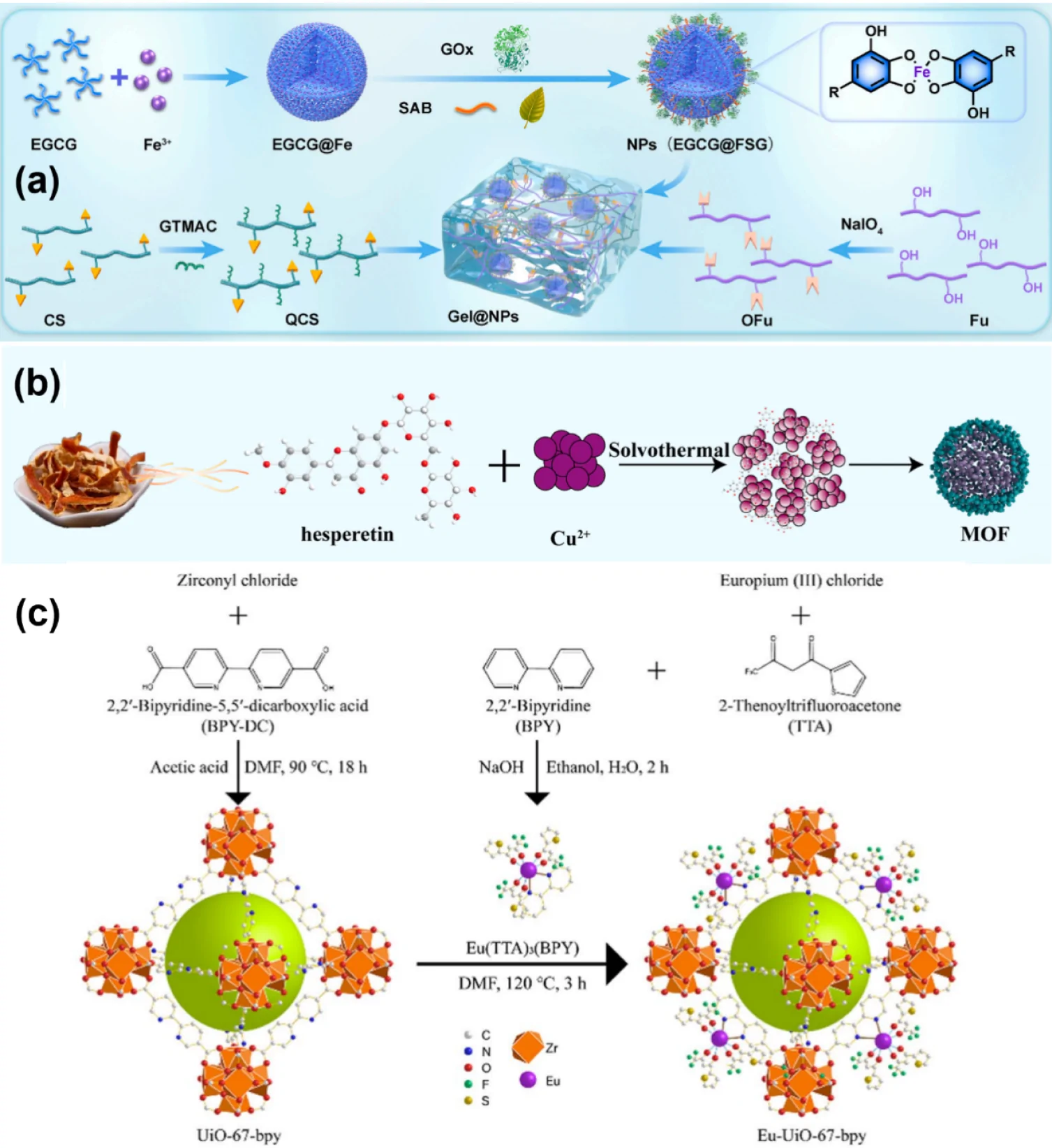
(a) Schematic representation illustrating the design strategy for hydrogel dressings incorporating EGCG@FSG. Reproduced with permission.^33^ Copyright 2025, Elsevier. (b) Schematic diagram detailing the synthesis process of CuHspMOF. Reproduced with permission.^20^ Copyright 2024, Elsevier. (c) Synthetic pathway schematic for Eu-MOF. Reproduced with permission.^71^ Copyright 2022, BMC.

#### 2.1.2 Representative Systems

The physical embedding approach is broadly applicable across a wide range of metal complexes and hydrogel matrices (see Table 1).

**Table 1:** Representative Physically Embedded Systems and Properties

**Representative System**	**Metal Ion**	**Ligand Type**	**Complex Type**	**Hydrogel Matrix**	**Fabrication Method**	**Key Functionality**	**Reference**
EB@CuHsp Hydrogel	Cu^2+^	Hsp	Cu(II)-based Bio-MOF	ε-Polylysine Methacrylate	A combination of physical mixing and photocuring (Figure 2b)	pH-responsive colorimetric monitoring with synergistic antibacterial action	^20^
RuMePhen/SPS Hydrogel	Ru^2+^	MePhen	Tris-bidentate homoleptic Ru(II) complex	Gelatin	Visible-light-initiated radical crosslinking	Synergistic photo-crosslinking capability and antibacterial activity	^21^
EGCG@FSG@QCS/OFu Hybrid Hydrogel	Fe^3+^	Polyphenol	Metal-Polyphenol Coordination Network	QCS/OFu	Direct mixing of EGCG@FSG nanoparticles into QCS/OFu precursor, followed by encapsulation via gelation	Synergistic multi-bioactivity response	^33^
Eu-UiO-67-bpy Hydrogel	Eu^3+^	TTA and bpy	Lanthanide MOF	GelMA	Physical mixing followed by photocuring (Figure 2c)	Dual-modal fluorescence-CT imaging capability with anti-diffusion stability	^71^
[Pt(tpy)NCS]^+^/Cl^–^@PVA Hydrogel	Pt^2+^	tpy, −NCS	Pt(II)-tpy complex	PVA	Immersion of PVA hydrogel in [Pt(tpy)NCS]^+^/Cl^–^ solution for 12 h	Mercury ion-induced 1D supramolecular assembly	^72^
Cu-MOFs/Agarose Hydrogel	Cu^2+^	BTC	Cu(II)-MOFs	Agarose	Direct dispersion of Cu-MOFs in agarose solution	High-sensitivity H_2_S colorimetric detection, rapid response, visible color change, portability	^73^
Zn-L@SA Hydrogel	Zn^2+^	Amide-functionalized dibenzimidazole ligand	Mononuclear zinc complex	SA/PVA composite matrix	Ca^2+^ crosslinking of SA	Highly selective fluorescent recognition and ultra-sensitive detection of fluoroquinolone antibiotics	^74^
MOF-1@HA/CMCS Hydrogel	Cd^2+^/Zn^2+^	N-donor polycarboxylate ligands with auxiliary N-ligands	bimetallic MOF	HA/CMCS	Drug-loaded MOF was ultrasonically adsorbed, then directly mixed into a CMCS solution and crosslinked with HA to form the hydrogel	Controlled drug release and genetic modulation	^75^

#### 2.1.3 Examples

Fe-Polyphenol Nanocomposite Hybrid Hydrogel^33^

**Synthesis:** The hydrogel is constructed via a multi-step self-assembly and cross-linking process. Initially, epigallocatechin gallate (EGCG) and Fe^3+^ ions self-assemble to form metal-polyphenol nanoparticles (EGCG@Fe) (Figure 2a). These nanoparticles are subsequently loaded with salvianolic acid B (SAB) and glucose oxidase (GOx), yielding the functionalized EGCG@FSG nanoparticles. Separately, quaternized chitosan (QCS) and oxidized fucoidan (OFu) are prepared and subsequently cross-linked through Schiff base reactions to form the hydrogel network. Finally, the EGCG@FSG nanoparticles are embedded within this hydrogel matrix, producing the hybrid system (Gel@NPs).

**Functionality:** Antibacterial: QCS and Fe^3+^ synergistically disrupt bacterial membranes, effectively eliminating pathogens such as *S. aureus* and *E. coli*. Antioxidant: Phenolic hydroxyl groups in EGCG mitigate oxidative stress associated with ulcerated tissues. Hypoglycemic: GOx catalyzes glucose oxidation, reducing local glucose concentrations. Anti-inflammatory: SAB promotes M2 macrophage polarization, suppresses pro-inflammatory cytokines (e.g., TNF-α), upregulates anti-inflammatory mediators (e.g., IL-10), and activates VEGF signaling pathways to increase angiogenesis and collagen deposition. Responsive release: In the acidic microenvironment of diabetic foot ulcers (pH 5.5), dynamic Schiff base bonds are cleaved, enabling controlled release of the nanoparticles and therapeutic agents.

#### 2.1.4 Advantages, Limitations, and Applications

This strategy is conceptually straightforward and broadly applicable to a wide range of coordination systems, including MOFs as well as mono- and dinuclear metal complexes. In certain cases, gel formation occurs spontaneously through simple processing steps; for instance, Cu-based MOFs can be incorporated into agarose matrices to form stable hydrogels via direct mixing followed by cooling.^73^ The intrinsic porosity and three-dimensional network of hydrogels can further improve the functional performance of the embedded complexes. This effect has been exemplified by poly­(vinyl alcohol) (PVA) hydrogels, which significantly amplify analytical signals, enabling trace-level detection.^72^

Despite these advantages, physical embedding is associated with several inherent limitations. The non-covalent nature of the interactions between the complexes and the hydrogel matrix increases the risk of leaching, particularly during prolonged operation. For example, the *in vivo* stability of Cd–Zn MOFs incorporated within hyaluronic acid/carboxymethyl chitosan (HA/CMCS) hydrogels has yet to be conclusively established.^76^ Moreover, the loading capacity is intrinsically constrained by the porosity of the hydrogel network. Systems such as Pt complexes in PVA hydrogels and Cu–tannic acid (Cu–TA) in silk fibroin matrices exhibit limited loading thresholds, beyond which aggregation becomes pronounced.^72,77^ Even when ultrasonic dispersion is employed, complete suppression of aggregation is difficult to achieve, often resulting in heterogeneous distribution throughout the hydrogel matrix.

Physically embedded hydrogel systems are particularly well-suited for applications requiring rapid responsiveness. Mercury-responsive test strips based on PVA hydrogels, for example, enable swift colorimetric or fluorescence-based detection, supporting prompt diagnostic decision-making.^72^ Similarly, HA/CMCS hydrogels have been shown to facilitate controlled drug release while limiting the diffusion of free heavy metal ions, mitigating associated toxicity.^76^ In another application, Cu-MOF-integrated epsilon-poly-l-lysine (EPL) hydrogels exhibits effective bactericidal activity through photothermal mechanisms, highlighting their potential as antibacterial wound dressings.^20^ However, the long-term structural and functional stability of such systems remains a critical challenge. This limitation is particularly significant in chronic pathological settings, such as non-healing diabetic wounds, where sustained therapeutic performance is essential.

### 2.2 Chemical Grafting

#### 2.2.1 Principles

Chemical grafting entails the covalent attachment of metal complexes to hydrogel polymer networks, resulting in stable integration and molecular-level dispersion. This synthetic strategy is based on ligand functionalization followed by copolymerization. Ligands within the metal complexes are modified to introduce polymerizable groups, which are subsequently copolymerized with hydrogel monomers to form covalently linked networks.

Characterization should confirm both covalent incorporation and preservation of the coordination sphere. FTIR band shifts, including those assigned to C=O, Cu–O, or O–H modes, may suggest an interaction but do not alone prove grafting. Evidence should combine conversion or coupling assays with NMR or mass spectrometry where applicable, XPS or elemental analysis for metal speciation and content, extraction/leaching tests, and rheology or swelling measurements.

#### 2.2.2 Representative Systems

Some metal-coordinated hydrogels are synthesized via chemical grafting strategies, and their corresponding properties are summarized in Table S1.^78–82^

#### 2.2.3 Examples

CEG–Fe^3+^ hydrogel^80^

**Synthesis:** The CEG–Fe^3+^ hydrogel extends ligand grafting to a polysaccharide-based wound-dressing candidate. Ethylenediamine is first coupled to carboxymethyl cellulose via EDC/NHS chemistry, after which gallic acid is grafted onto the aminated polymer through a second amide-coupling reaction. The reported ethylenediamine grafting rate is 14.84%, and the gallic-acid substitution degree is 32.7%. Fe^3+^ is subsequently introduced to form pH-dependent pyrogallol–Fe^3+^ coordination crosslinks.

**Functionality:** The hydrogel exhibits a self-healing efficiency of approximately 88%, pig-skin adhesion strengths of 6.98 and 11.44 kPa at pH 7 and 9, respectively, a cell-survival rate of 84%, a hemolysis ratio below 5%, and antibacterial and antioxidant activities. These data support its potential as an adhesive wound dressing, but they do not yet establish therapeutic wound closure because no *in vivo* wound-healing model has been reported. Long-term Fe release, tissue response, and network stability in protein-containing physiological media also warrant evaluation.

#### 2.2.4 Advantages, Limitations, and Applications

Covalent integration provides the tightest control over stoichiometry and minimizes passive leaching of an intact complex. It is particularly useful when the complex is intended to remain part of the network until a defined chemical or photochemical event occurs. This has been exemplified in ruthenium-based hydrogel systems, where controlled degradation can be achieved within 3-D networks without compromising overall structural coherence.^40^ Beyond stability, covalent integration supports multifunctionality, allowing the coexistence of strong interfacial adhesion and preserved optical properties, such as fluorescence.^83^ Moreover, material properties can be systematically tailored by adjusting the grafting density through controlled variation of the metal complex concentration, resulting in improvements in mechanical parameters, including stiffness and elastic modulus.^40,84^

Despite these advantages, chemical grafting strategies are often associated with considerable synthetic and translational challenges. Ligand functionalization must preserve metal coordination while providing a polymer-reactive group; reaction conversion, residual catalyst or monomer, and metal speciation must be controlled batch-to-batch. The requirement for sequential steps, including metal complex synthesis, hydrogel network formation, and post-loading of therapeutic agents, introduces procedural complexity that elevates production costs and restricts large-scale fabrication.^85^ Furthermore, retention should not be equated with biological safety: cleavage products may diffuse more readily than the parent crosslinker, and incomplete coupling generates extractable species. In addition, the incorporation of transition metal species necessitates biocompatibility assessments, as potential cytotoxicity and long-term metal ion exposure must be carefully evaluated through *in vitro* and *in vivo* studies.^20,76,85^

The successful implementation of chemically grafted systems in real-world applications requires careful matching of material characteristics with specific functional demands. The small number of demonstrated biomedical examples also highlights key considerations for future development. In wound dressing applications, gallic acid-grafted carboxymethyl cellulose hydrogels coordinated with Fe^3+^ have been engineered, exhibiting self-healing properties, antibacterial and antioxidant activities, and robust tissue adhesion (11.44 kPa on pigskin).^80^ In diabetic wound repair, dopamine-grafted oxidized hyaluronic acid hydrogels with Fe^3+^ coordination facilitate pH-responsive metformin release through dynamic imine and metal–polyphenol bonds, achieving rapid hemostasis (<10 s), photothermal antibacterial efficacy (≈100%), and anti-inflammatory effects that promote scarless healing.^81^

### 2.3 Coordination Crosslinking

#### 2.3.1 Principles

Coordination crosslinking represents a fundamental strategy for integrating metal complexes into hydrogel networks. This approach relies on the construction of three-dimensional polymeric architectures through reversible metal-ligand interactions, enabling a balance between mechanical integrity and stimuli-responsive functionality.

The performance of coordination-crosslinked hydrogels arises from the dynamic nature of metal–ligand bonds formed between metal ions and ligand functionalities tethered to polymer chains, including carboxylate, catechol, and sulfonate groups. Specifically, the catechol group, owing to its *o*-diphenol dihydroxy chelating structure, displays strong coordination interaction and good stability with many transition metal ions. The carboxyl group coordinates in a monodentate or bidentate mode, with moderate binding strength, while the sulfonic acid group possesses relatively weaker stability. This trend in binding affinities is capable of regulating the relevant properties of hydrogels. Under applied mechanical stress, these coordination bonds dissociate and subsequently re-form upon stress release, imparting self-healing characteristics to the material.^66^ The spatial arrangement and chemical nature of the coordination sites play a critical role in governing network behavior. Ligands may be covalently grafted onto polymer backbones, such as the attachment of 2,6-bis­(1,2,3-triazol-4-yl)­pyridine (btp) moieties to tetrameric PEG, or derived directly from functional groups inherent to the polymer matrix, as exemplified by the carboxyl groups present in HA.^18,67^

A combination of analytical techniques is typically employed to elucidate structural features and mechanical performance. X-ray photoelectron spectroscopy (XPS) is commonly used to confirm coordination bond formation, as demonstrated by an S/Zr atomic ratio close to 0.5 in Zr^4+^–sulfonate systems.^13^ Concurrently, mechanical reinforcement is evidenced by quantitative measurements, including fracture strains approaching 400% and elastic modulus values on the order of 63.7 kPa.^84^

#### 2.3.2 Representative Systems

Some metal-coordinated hydrogels are fabricated using coordination crosslinking strategies, and their corresponding properties are summarized in Table S2.^13,32,66,67^

#### 2.3.3 Examples

(1) Sulfonate-Zr^4+^ Coordination Hydrogel^13^

**Synthesis:** A copolymeric hydrogel based on acrylamide (AAm) and 2-acrylamido-2-methylpropanesulfonic acid (AMPS) is first prepared through free-radical polymerization in the presence of chemical crosslinkers (Figure 3a). The resulting hydrogel is subsequently swollen and subjected to sequential immersion in an aqueous ZrOCl_2_ solution, promoting the formation of coordination interactions between sulfonate groups and Zr^4+^ ions. Excess ions are removed by thorough rinsing with deionized water.

**Figure 3:**
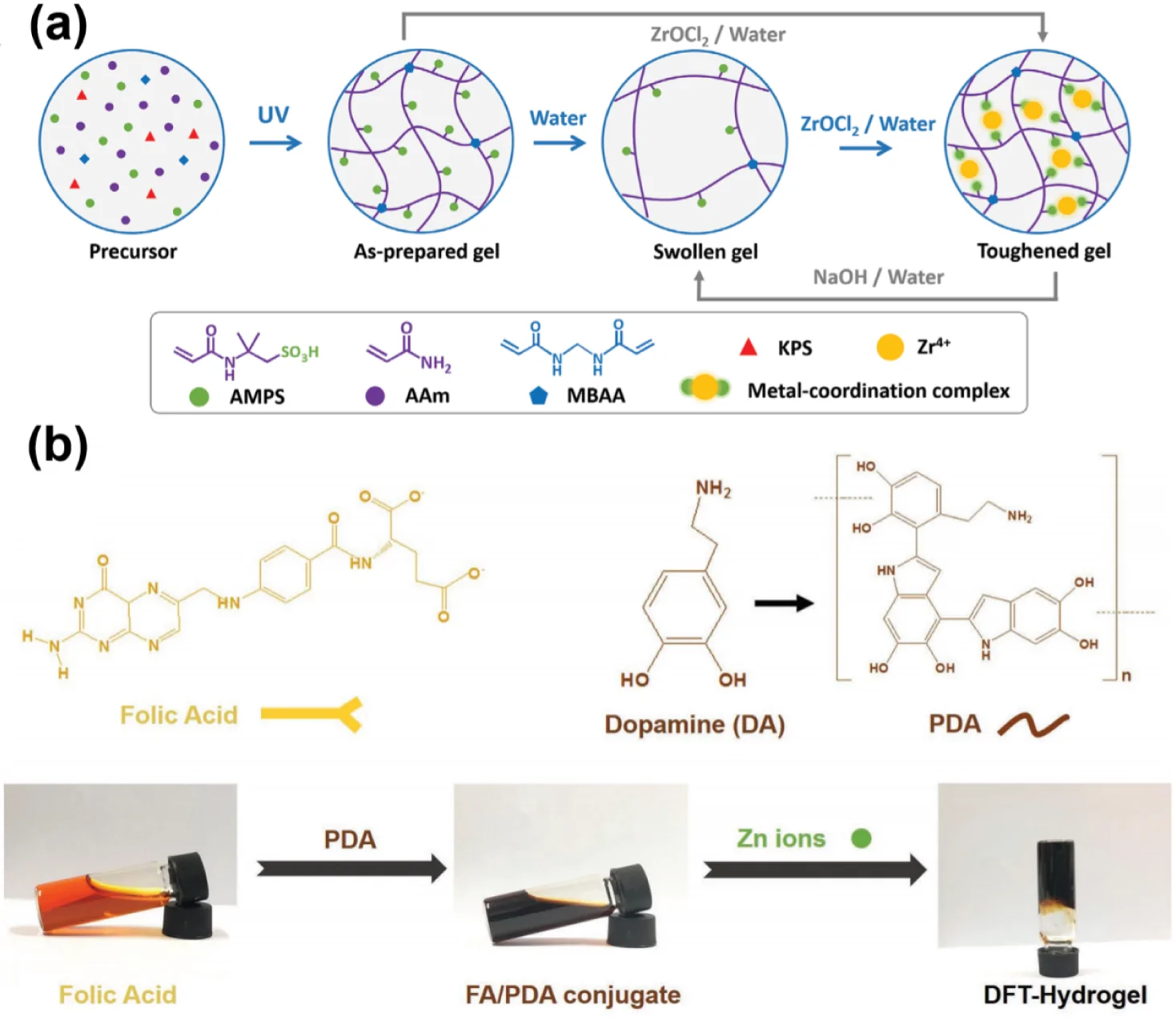
(a) Schematic representation of P­(AAm-*co*-AMPS) hydrogel synthesis and reversible transitions between swollen/toughened states. Reproduced with permission.^13^ Copyright 2020, John Wiley and Sons. (b) The molecular structures and the photos of hydrogel synthesis. Reproduced with permission.^32^ Copyright 2019, John Wiley and Sons.

**Functional Properties:** The reversible nature of sulfonate–Zr^4+^ coordination imparts dynamic mechanical behavior, allowing the material to reversibly transition between a highly swollen, brittle state and a mechanically robust, tough state. This tunable mechanical performance, combined with its responsiveness to external stimuli, underpins its potential utility in a range of applications, including soft actuators, grippers, connectors, sealing materials, and biomedical implantable devices.

(2) Zn^2+^-FA/PDA Hydrogel^32^

**Synthesis:** Folic acid is first dissolved in an alkaline aqueous medium, where chain extension is induced (Figure 3b). Subsequent addition of dopamine initiates polymerization, leading to the formation of a polydopamine (PDA) network. Prior to the crosslinking step, pre-synthesized C/ZnO nanoparticles are introduced and uniformly dispersed into the precursor solution. The introduction of Zn^2+^ ions then rapidly crosslinks the system through coordination interactions, involving chelation between Zn^2+^ and the carboxyl groups of folic acid as well as the catechol functionalities of PDA. This one-step coordination process simultaneously encapsulates the nanoparticles within the forming network. This coordination-driven network formation occurs within seconds under ambient conditions.

**Functional Properties:** The resulting composite hydrogel exhibits light-activated antibacterial and wound-healing capabilities. Upon exposure to 660 nm red light and 808 nm near-infrared irradiation, the uniformly embedded C/ZnO nanoparticles generate reactive oxygen species and induce localized hyperthermia reaching approximately 55 °C, resulting in efficient bacterial membrane disruption with antibacterial efficiencies exceeding 99.9% against *Staphylococcus aureus* and *Escherichia coli*. Similarly, sustained release of Zn^2+^ over 12 days inhibits bacterial proliferation. Concurrently, Zn^2+^ promotes fibroblast proliferation and increases the expression of collagen (COL-I/III) and matrix metalloproteinase-2 (MMP-2), thus accelerating tissue regeneration.

#### 2.3.4 Advantages, Limitations, and Applications

Coordination-crosslinked hydrogels exhibit pronounced multifunctionality, largely arising from their tunable mechanical characteristics. For example, the mechanical modulus of P­(AAm-*co*-AMPS) hydrogels can be systematically adjusted by varying the concentration of Zr^4+^ ions, enabling precise control over network stiffness.^13^ Further, the incorporation of biocompatible polymer components such as hyaluronic acid (HA) and dopamine (DA), together with the use of metal ions of comparatively low toxicity, supports their suitability for biomedical applications.^18^

Despite these advantages, several constraints are inherent to metal–ligand coordination chemistry. Sulfonate-based ligands display strong selectivity toward Zr^4+^ ions, while exhibiting limited coordination efficiency with alternative metal species.^13^ Iron-based systems present further challenges: Fe^2+^ is readily oxidized to Fe^3+^ in aqueous environments, which can destabilize the hydrogel network and necessitate strictly controlled or anhydrous conditions.^67^ Moreover, Fe^3+^–catechol coordination complexes are susceptible to dissociation under alkaline conditions, restricting their applicability in basic environments.^66^

A range of application domains has been explored for these materials. In biomedical contexts, Zn^2+^–PDA/FA hydrogels have demonstrated improved wound healing in infected models, achieving 95.7% wound closure within 10 days, compared with 70.5% in untreated controls. This performance is attributed to synergistic effects arising from dual-light-induced reactive oxygen species generation and sustained Zn^2+^ release.^32^ Similarly, Ca^2+^–bisphosphonate (BP) hydrogels have been shown to replicate aspects of natural bone mineralization and promote bone regeneration, as confirmed by micro-computed tomography analysis of bone volume fraction (BV/TV).^86^ In the field of soft robotics, Zr^4+^–sulfonate hydrogels have been developed as high-force tubular actuators capable of exerting grasping forces exceeding 150 N, corresponding to approximately 4000 times their own weight; however, their practical deployment is constrained by sensitivity to alkaline environments.^13^ For sensing and optical applications, Eu^3+^/Tb^3+^-doped carbon dot (CD) hydrogels exhibit temperature-dependent luminescence shifts with strong linear correlations (R^2^ > 0.99), although comprehensive biocompatibility validation remains outstanding.^84^ Several challenges continue to impede broader translation of coordination-crosslinked hydrogels. These include the need for more extensive biocompatibility assessments, unresolved issues related to clinical translation, such as the risk of cumulative metal ion toxicity (e.g., Ag^+^)^18^ and limitations in industrial scalability arising from high production costs and complex fabrication processes.^18,84^

### 2.4 *In Situ* Synthesis

#### 2.4.1 Principles

Conventional approaches that rely on incorporating preformed metal complexes into hydrogel precursors frequently suffer from drawbacks such as particle aggregation, weak interfacial interactions, and gradual leaching, all of which compromise the structural and functional stability of the resulting materials (Figure 4a).^8^ *In situ* synthesis strategies address these limitations by generating metal complexes directly within the hydrogel network, enabling molecular-level dispersion and stronger interfacial integration.

**Figure 4:**
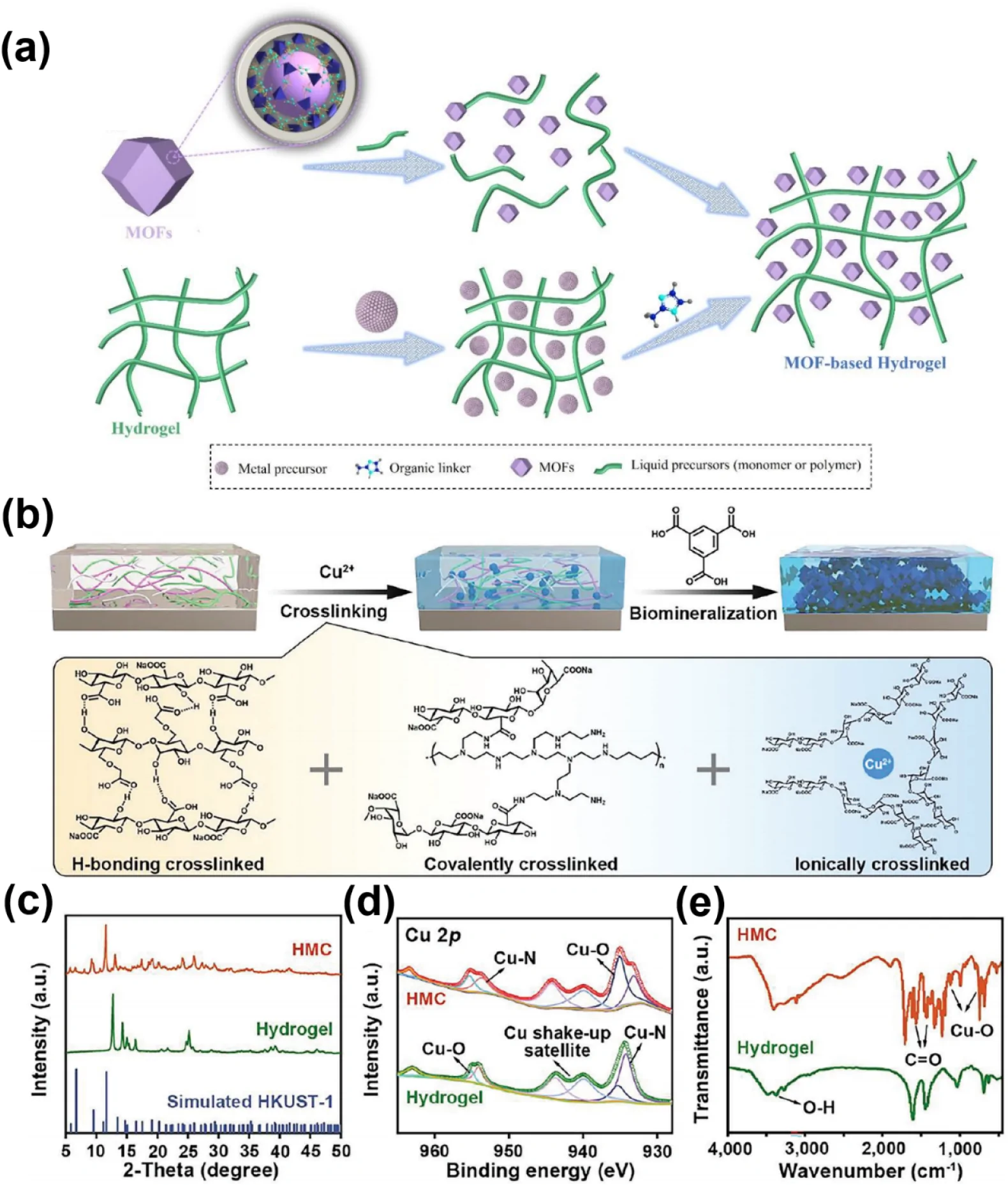
(a) Schematic of MOF-based hydrogel preparation via simple mixing and *in situ* growth. Reproduced with permission.^8^ Copyright 2023, Royal Society of Chemistry. (b–e) Synthesis and characterization of Cu-MOF-biopolymer hydrogel. Reproduced with permission.^88^ Copyright 2023, John Wiley and Sons. (b) HMC preparation procedure; (c) XRD patterns comparing hydrogel coating/HMC to standard HKUST-1; (d) high-resolution Cu *2p* XPS spectra; (e) FTIR spectra of hydrogel coating/HMC.

A commonly employed *in situ* route follows a two-step sequence. Initially, metal ions interact with functional groups present in the hydrogel matrix to establish anchoring sites through coordination interactions. Subsequent introduction of appropriate ligands induces nucleation and controlled growth of the metal complexes within the network.^87^ A representative example is provided by lanthanide-based luminescent hydrogels, in which Eu^3+^/Tb^3+^ complexes are immobilized within the polymer framework during network formation via copolymerization of acrylamide (AAm) with N,N′-methylenebis­(acrylamide) (MBAA), allowing direct incorporation during polymerization.^83^

X-ray diffraction (XRD) is used to verify the crystal structure. SEM and transmission electron microscopy (TEM) are employed to observe crystal size and distribution. Spectroscopic techniques, such as ultraviolet-visible (UV–vis) and FTIR, are used to elucidate the chemical composition and coordination environment of the materials.^88^

#### 2.4.2 Representative Systems

Some metal-coordinated hydrogels are fabricated *via in situ* synthesis strategies, and their corresponding properties are summarized in Table S3.^83,84,87–91^

#### 2.4.3 Typical *In Situ* Synthesis

Cu-MOF-Biopolymer Hydrogel^88^

**Synthesis:** Sodium alginate (SA), CMC, and polyethylenimine (PEI) are first combined to form a homogeneous biopolymer mixture, which is subsequently applied as a coating onto a substrate to generate a hydrogel layer (Figure 4b–e). The coated layer is then immersed in an aqueous Cu­(II) acetate solution, inducing ionic crosslinking within the polymer network. Following this step, an ethanolic solution of 1,3,5-benzenetricarboxylic acid (H_3_BTC) is introduced, triggering the *in situ* nucleation and growth of HKUST-1 within the hydrogel matrix. This process results in the formation of a compact, brick-and-mortar-like composite coating.

**Functionality:** The resulting hydrogel coating exhibits combined antifouling and corrosion-resistant behavior. The hydrophilic nature of the gel, together with a balanced surface chemistry, effectively suppresses adhesion of proteins, bacteria (*Pseudomonas aeruginosa*), and algae (*Chlorella*). Cu^2+^ ions released from the MOF disrupt microbial cell membranes, imparting broad-spectrum antimicrobial activity. The integrated structure also serves as a physical barrier against corrosion; specifically, controlled Cu^2+^ release inhibits biofilm formation and significantly reduces pitting corrosion under acidic conditions. Similarly, the inherent porosity of the MOF enables the incorporation of functional agents, such as natural rosin extracts, extending the protective performance of the coating over prolonged periods.

#### 2.4.4 Advantages, Limitations, and Applications

One significant advantage of MOF–hydrogel composites is the enhanced interfacial bonding arising from strong coordination or hydrogen-bonding interactions between the embedded complexes and the polymeric network, which effectively suppresses leakage; for example, UiO-66@gelatin exhibits a high Pb^2+^ adsorption capacity of 914 mg/g.^92^ Another key benefit is functional synergy, where the hydrogel acts as a flexible, hydrated substrate while the incorporated complexes confer catalytic or sensing functionalities; MOF-808@hydrogel, for instance, combines heavy metal adsorption with potential activity toward nerve agent degradation.^92^

Despite these advantages, several challenges persist. Certain MOFs, such as ZIF-8, require stringent pH and temperature control due to their propensity to decompose under acidic conditions, which limits operational robustness.^89^ Moreover, high loadings of complexes can detrimentally impact the mechanical integrity of the composite; at 60 wt % loading compared to 20 wt %, pronounced reductions in tensile stress and Young’s modulus are observed under low-strain conditions.^93^ Furthermore, scalability remains challenging as precise parameter control during *in situ* synthesis complicates large-scale production.^94^

In the context of environmental remediation, MOF–hydrogel systems leverage the high density of adsorption sites inherent to MOFs alongside the increased mass transfer afforded by the hydrated polymer matrix. For example, ZIF-8@chitosan displays a CO_2_ adsorption capacity of 1.21 mmol/g,^95^ and Al-based MIL-121@alginate hydrogels show promising potential for wastewater treatment, although their long-term cycling stability requires further evaluation.^96^ The vulnerability of MOF structures in acidic environments underscores the need for protective surface modifications to preserve framework integrity.^97^ In biomedical applications, MOF–hydrogel composites integrated with photothermal and photodynamic therapy (PTT/PDT) modalities have demonstrated synergistic antibacterial, anti-inflammatory, and pro-angiogenic effects when used as wound dressings, improving wound healing outcomes.^98^ In drug delivery, these composites enable controlled and sustained release profiles; for instance, the CMC/5-FU@MOF-5 hydrogel facilitates regulated release of the anticancer agent 5-fluorouracil (5-FU).^99^

### 2.5 Critical Comparison of the Four Integration Strategies

The four strategies address different design problems and should be selected according to the intended mechanism of action.^3–5,8,18,40,69,87–90^ Physical embedding is the simplest and often the most scalable route for short-residence depots, dressings, and sensing, but it is most susceptible to sedimentation and burst release. Covalent grafting provides high retention and a defined molecular connection, yet introduces synthetic steps and may prevent uptake or ion exchange. Coordination crosslinking offers injectability, energy dissipation, and reversible response, while remaining sensitive to dilution and biological competitors. *In situ* growth improves particle dispersion and interfacial contact, but nucleation and residual precursor control become additional critical attributes.

These tendencies are conditional rather than absolute. A physically embedded particle may strengthen a network; a covalently tethered complex may still release rapidly after a labile linker is cleaved; and an *in situ* process may proceed at mild room-temperature conditions. Release may be governed by diffusion, reversible coordination, network erosion, particle dissolution, or a changing combination of these processes.^4,5,31,40,89,95,100^ The comparative criteria summarized in Table S4 reflect these considerations.

### 2.6 Structure–Property–Function Relationships

**Metal and Ligand Chemistry.** Metal identity dictates preferred geometry, ligand-exchange kinetics, redox activity, and the consequences of release. Oxidation state must be measured rather than inferred from the precursor, particularly for Fe, Cu, and Mn systems. Ligand denticity, protonation, and hard–soft matching then determine whether the complex remains intact, exchanges with proteins, or serves as a dynamic junction. Molecular studies of Ni^2+^–nitrogen networks illustrate that bond geometry and cooperative rupture, not nominal bond strength alone, govern macroscopic toughness.^101,102^

**Network Architecture.** The number and lifetime of metal–ligand junctions determine percolation, modulus, stress relaxation, and self-healing, while the polymer mesh governs solvent and solute transport. Increasing metal content can raise crosslink density until ligand saturation, precipitation, or network heterogeneity reverses the trend. Consequently, mechanical testing should be combined with spatial mapping and metal speciation across the same composition series.^103,104^

**Release and Therapeutic Function.** The apparent release profile is the combined result of diffusion through the mesh, metal–ligand dissociation, particle dissolution, polymer erosion, and rebinding to the matrix or biological molecules. A single cumulative-release curve cannot deconvolute these processes. Total metal, labile metal, intact complex, ligand, and co-loaded drug should be quantified separately when mechanistically distinct.^105,106^

**Design Implications.** An antibacterial dressing may benefit from an initially labile fraction followed by sustained release, whereas a piezoelectric or imaging scaffold may require metal retention with negligible leaching. An anticancer depot may deliberately couple acidic dissolution to cargo release, but only if the same trigger does not produce off-target exposure. The appropriate coordination lifetime is therefore defined by the clinical task rather than by maximized stability.^107,108^

## 3 Biomedical Applications

### 3.1 Anti-Inflammatory Effect of Metal-Coordinated Hydrogels

#### 3.1.1 Mechanism

The progression of osteoarthritis (OA) is strongly associated with oxidative stress and chronic inflammation within the joint microenvironment.^109–112^ Certain metal ions (e.g., Mg^2+^, Ag^+^) display robust anti-inflammatory and antioxidant activities via the inhibition of pivotal inflammatory signaling pathways such as NF-κB and TLRs.^113^ Cu^2+^, Sr^2+^, and Co^2+^ remodel the local tissue microenvironment to facilitate angiogenesis and osteogenesis.^114,115^ Coordination between metal ions and hydrogel matrices reinforces the structural and mechanical integrity of the hydrogel network, enabling the sustained and localized release of therapeutic metal ions. In certain hydrogel systems, coordination with metal ions can induce piezoelectric properties. This electromechanical stimulus activates voltage-gated calcium channels to modulate ATP oscillation and cytoskeletal rearrangement, thereby upregulating the expression of cartilage-specific proteins. Additionally, they facilitate acetylcholine release from vagal nerve terminals, which collectively suppresses NF-κB nuclear translocation and NLRP3 inflammasome activation. This cascade further downregulates the production of pro-inflammatory cytokines including TNF-α, IL-6 and IL-1β, ultimately eliciting potent anti-inflammatory responses and facilitating cartilage regeneration.^116,117^

#### 3.1.2 Examples

PBT-Gel Piezoelectric Hydrogel^118^

**Material Design:** The hydrogel system was developed through coordination interactions between Ga^3+^ and HA, resulting in the formation of a Ga-HA network. This matrix was subsequently embedded with PDA-modified barium titanate (PBT) nanoparticles, yielding a composite hydrogel (PBT-Gel) with enhanced piezoelectric and bioactive properties (Figure 5a–c).

**Figure 5:**
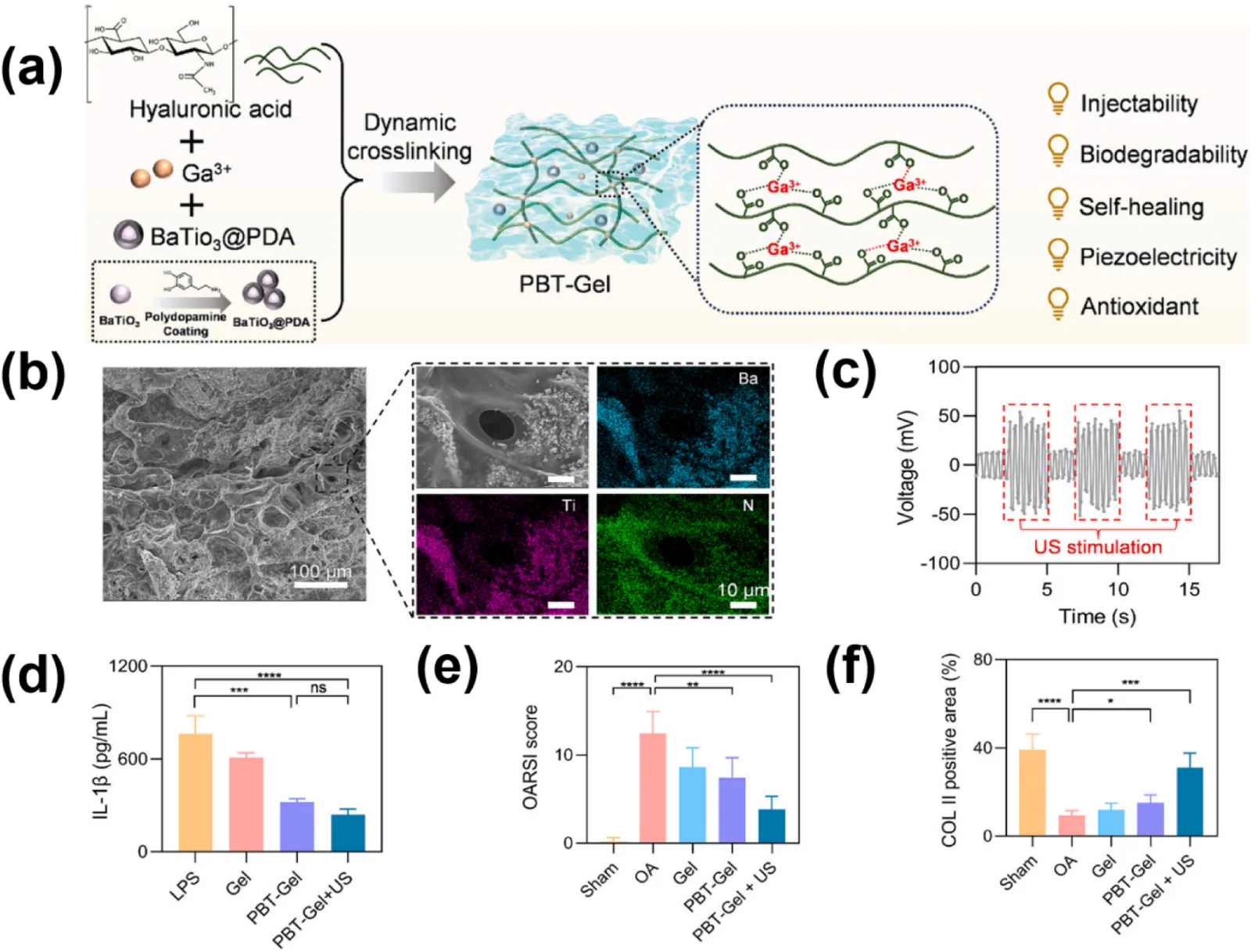
(a) Schematic diagram for the preparation of PBT-Gel hydrogel. (b) SEM images and EDS surface-scan element distribution of PBT-Gel (n = 3). Scale bar = 100 μm. (c) Mechanical-electric response of PBT-Gel before and after US stimulation. (d) *In vitro* anti-inflammation of PBT-Gel hydrogel by IL-1β ELISA test (n = 3). *P < 0.05, **P < 0.01, ***P < 0.001, ****P < 0.0001, ns indicates no significant difference. (e) OARSI histological scoring of the generated tissues in different groups (n = 5). (f) Quantitative analysis of immunohistochemical staining of Col II (n = 5). *P < 0.05, **P < 0.01, ***P < 0.001, ****P < 0.0001, ns indicates no significant difference. Reproduced with permission.^118^ Copyright 2025, Elsevier.

**Mechanism:** When stimulated by ultrasound (US), the PBT-Gel produces localized piezoelectric signals, which activate the TGF-β/Smad pathway and thus facilitates cartilage regeneration.^118,119^ The anti-inflammatory effect of this gel is attributed to its ROS-scavenging activity, which alleviates oxidative stress in the joint microenvironment.

**Performance:** (1) Cartilage regeneration: Histological analyses revealed that the PBT-Gel + US treatment group exhibited substantial restoration of the cartilage matrix. (2) Extracellular Matrix (ECM) maintenance: Type II collagen (Col II) and SRY-related HMG-box 9 (SOX9) are key regulators of ECM integrity in cartilage. Immunohistochemical staining demonstrated minimal Col II and SOX9 expression in the saline-treated group, while the PBT-Gel group showed strong, continuous positive staining for both markers. (3) Inflammation suppression: The PBT-Gel + US group displayed no signs of synovial hyperplasia and had the lowest synovitis score among osteoarthritic rats (Figure 5d–f).

#### 3.1.3 Performance Comparison

The anti-inflammatory mechanisms and performance of representative metal-coordinated hydrogels are summarized in Table S5.^77,118,120,121^

#### 3.1.4 Critical Appraisal

Most reports describe favorable histology or short-term biomarker changes in rodent models, but few isolate the metal contribution from that of the hydrogel, ultrasound, or a co-delivered drug.^5,70,77,118,120,121^ Follow-up is often too short to evaluate recurrent synovitis, cartilage durability, or metal redistribution. The ^177^Lu system additionally requires dosimetry and radiometabolite clearance rather than being evaluated as a conventional antioxidant hydrogel. Large-animal joint mechanics, repeated dosing, and comparison with intra-articular hyaluronic acid or corticosteroid treatment are needed before a translational advantage can be claimed.

### 3.2 Anticancer Effect of Metal-Coordinated Hydrogels

#### 3.2.1 Mechanism

By utilizing the intrinsic bioactivity of metal ions, targeted sustained release via hydrogel delivery, and the system’s regulation of the tumor microenvironment (TME), the synergy between metal complex and hydrogel can improve anticancer efficacy. Transition metal ions can mediate chemodynamic therapy (CDT), i.e., catalyzing Fenton or Fenton-like reactions in the acidic TME rich in hydrogen peroxide to generate highly reactive hydroxyl radicals (·OH), thereby effectively eradicating tumor cells.^122^ They can also potentiate the therapeutic efficacy of photothermal therapy (PTT) and photodynamic therapy (PDT).^123^ In contrast, Cu^2+^ and Fe^3+^ are capable of directly inducing ferroptosis and cuproptosis in cancer cells.^124,125^ Ions such as Mn^2+^ can enhance the sensitivity of dendritic cells (DCs) to tumor DNA recognition.^122,126^ The integration of metals with hydrogels not only reinforces the mechanical properties of hydrogels and enables the sustained long-term release of ions, but also reduces systemic toxicity. For instance, Cu^2+^ can form potent cytotoxic complexes (CUET) with anticancer drugs, which exert certain anticancer effects but inevitably trigger systemic toxicity.^127,128^ The encapsulation by hydrogels can sequester these bioactive substances, thereby minimizing adverse reactions and improving biosafety.^129^ Meanwhile, the incorporation of paramagnetic ions (e.g., Mn^2+^) into hydrogels endows the resultant hydrogels with magnetic resonance imaging (MRI) functionality, allowing for the continuous *in vivo* tracking of the drug delivery system and dynamic evaluation of therapeutic outcomes.^130,131^

#### 3.2.2 Examples

Peptide-Mn^2+^ Coordinated Hydrogel^132^

**Material Design:** A dual-functional antitumor peptide (N-Pep) was coordinated with Mn^2+^ ions to construct a porous hydrogel (N-Pep-Mn gel). The resulting matrix was subsequently loaded with anti-PD-1 antibodies (αPD-1), yielding the composite therapeutic system αPD-1@N-Pep-Mn gel (Figure 6a–d).

**Figure 6:**
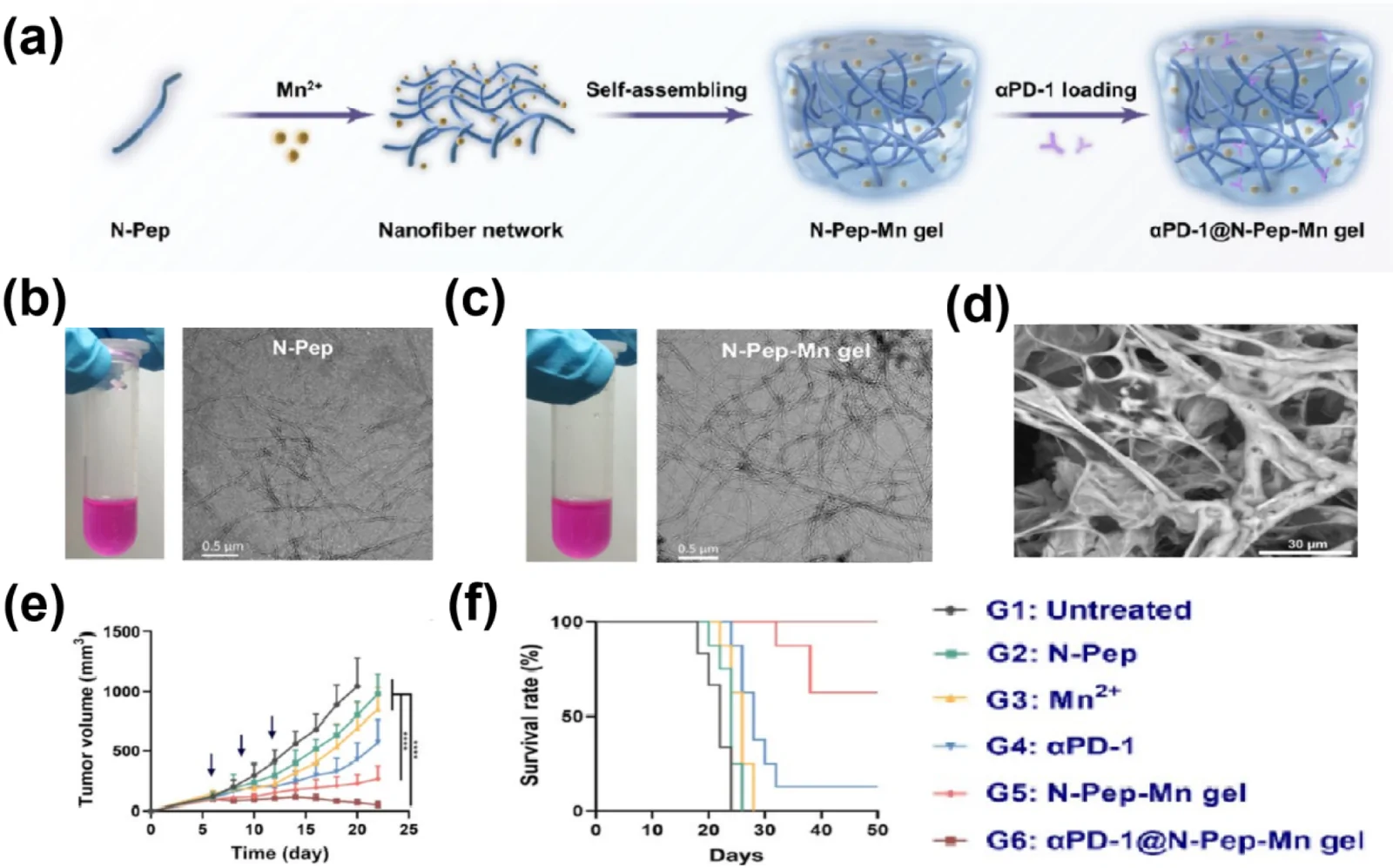
(a) Assembly process of αPD-1@N-Pep-Mn gel. (b, c) Photographs and transmission electron microscope (TEM) images of N-Pep and N-Pep-Mn gel in culture medium (scale bar: 0.5 μm). (d) Scanning electron microscope (SEM) images of N-Pep-Mn gel (scale bar: 30 μm). (e, f) Tumor growth curves and corresponding survival rate of mice after different treatments (G1, n = 6, G2–G6, n = 8). Reproduced with permission.^132^ Copyright 2025, American Chemical Society.

**Anticancer Mechanism:** Immune checkpoint blockade (ICB): The sustained release of αPD-1 antibodies prevents the interaction between PD-L1 on tumor cells and PD-1 on cytotoxic T lymphocytes (CTLs), restoring CTL activity and amplifying systemic antitumor immune responses in peripheral immune organs. Meanwhile, Mn^2+^ activates the cyclic guanosine monophosphate-adenosine monophosphate synthase (cGAS)-STING signaling pathway, leading to increased secretion of type I interferons (IFN-I), maturation of dendritic cells (DCs), and increased tumor DNA recognition sensitivity.

**Performance:** (1) Antitumor efficacy and survival: Treatment with the αPD-1@N-Pep-Mn hydrogel significantly inhibited tumor growth and prolonged survival in H22 tumor-bearing mice. In the control group, all six mice succumbed within 20 days, whereas the eight mice treated with αPD-1@N-Pep-Mn gel achieved a 100% survival rate at 50 days. (2) Induction of immune memory and prevention of tumor recurrence: The αPD-1@N-Pep-Mn hydrogel also elicited robust tumor-specific immune memory. Upon re-challenge with H22 tumor cells, rapid tumor progression was observed in control mice, while those previously treated with the hydrogel completely rejected tumor growth and remained tumor-free for over 60 days (Figure 6e, f).

#### 3.2.3 Performance Comparison

The anticancer mechanisms and performance of representative metal-coordinated hydrogels are summarized in Table 2.

**Table 2:** Metal-Coordinated Hydrogels for Cancer Treatment

**Material system**	**Metal category**	**Drug loaded**	**Cancer**	**Anticancer mechanism**	**Performance**	**Reference**
HD/BER/GOx/Cu Gel	Cu^2+^	1. BER	Breast cancer	1. Cuproptosis	The ratio of tumor volume was 40% compared to control group	^133^
		2. GOx		2. CDT		
N-Pep-Mn^2+^ Gel	Mn^2+^	anti-PD-1	Liver cancer	1. ICB	The 50-day survival rate of tumor-bearing mice was 100% in the experimental group vs 0% in the control group.	^132^
				2. Enhance the recognition of tumor cell DNA		
Lipo-Ru-HA Gel	Ru^2+^ complex	-	Melanoma	1. Ferroptosis	The survival rate of mice reached 100% 17 days after treatment	^134^
				2. PDT		
				3. PTT		
CP1-Pac-HA-CMCS Gel	Cu^2+^ complex	Paclitaxel	Thyroid cancer	Regulate the expression of tumor-associated genes	With the increase in drug concentration, the viability of cancer cells decreased significantly	^135^
Ce6- CuO_2_–MnO_2_–HHA Gel	1. CuO_2_ nanoparticles	Ce6	Breast cancer	1. CDT	1. Tumor cell survival rate as low as 11%	^136^
	2. MnO_2_ nanoparticles			2. PTT	2. Complete inhibition of tumor	
				3. PDT	growth	
ES-Cu^2+^-Alg Gel	ES-Cu	Galactose	Colorectal cancer	Cuproptosis	The median survival time of mice in the experimental group was increased to 42 days with no tumor recurrence, whereas all mices in the control group died within 26 days	^137^
Ni-CP-Pac-HA-CMCS Gel	[Ni(L)(D-CAM)(H_2_O)]_n_	Paclitaxel	Cervical cancer	Downregulate the expression of the HNF1A gene	the mRNA level of HNF1A was significantly down-regulated after treatment	^138^
CisPt-HA-βCD-PEI Gel	Cisplatin	Multiple drugs	Breast cancer	Targeted release of anticancer drugs	Dose-dependent cytotoxicity was elicited in the experimental group	^139^
PDL1-LA-Fe^3+^ Gel	Fe^3+^	anti-PDL1	Glioblastoma	1. Ferroptosis	A 90% prolongation of median survival time was observed in treated mice relative to the control group	^140^
				2. ICD		
				3. Enhance immune response		
Ru–PC-PEITC-Alg Gel	Ru-PC complex	PEITC	Breast cancer	1. PTT	Rapid tumor dissolution accompanied by a continuous reduction in volume without recurrence	^141^
				2. CDT		
Pt–Se–CS-Gel	PS@CS nanozyme	-	Melanoma	1. PTT	The tumor cell mortality rate reached 100% in the experimental group, in contrast to the sustained viability of tumor cells in the control group.	^142^
				2. CDT		
Cel-GA-Cu^2+^ Gel	Cu^2+^	1. Cel	Breast cancer	1. Cuproptosis	A tumor suppression rate of 91.24% and a distant metastasis inhibition rate of 88.05% were achieved	^143^
		2. GA		2. CDT		
PA-Cu-HPCS Gel	PA-Cu complex	V9302	Hepatocellular carcinoma	Cu^2+^ generate toxic products CuET to kill tumor cells	1. Significant reduction in cancer cell activity	^144^
					2. Tumor growth inhibition rate reaches 67.5%	
PTX–PDA-ALA-Fe^3+^-CNC Gel	Fe^3+^	Paclitaxel	Breast cancer	1. CDT	Reduction of cancer cell viability to 2.7% and induction of apoptosis at a rate of 33.05%	^145^
				2. Chemotherapeutic effect of PTX		
Mn^2+^-HA-DOPA Gel	Mn^2+^	Gambogic acid	Colon cancer	Inhibit the proliferation of tumor cells	Demonstration of excellent antitumor efficacy, with prolonged survival in mice	^146^

#### 3.2.4 Critical Appraisal

The most impressive tumor-control results generally emerge from multimodal formulations that combine a metal catalyst, a photosensitizer or drug, local heating, and immune checkpoint blockade.^5,70,123,132,136,141–144^ Such designs can be effective, but they make causal attribution challenging. Many studies depend on a single subcutaneous syngeneic model and report tumor shrinkage without long-term survival, recurrence, or systemic metal clearance. ROS-generating systems also have a therapeutic window: the same chemistry that damages tumor cells can injure wound margins and immune cells. Orthotopic and resection models, component-matched controls, blinded pathology, survival as a prespecified end point, and organ-level metal pharmacokinetics would provide stronger evidence than further increases in nominal multifunctionality.

### 3.3 Antibacterial Performance of Metal-Coordinated Hydrogels

#### 3.3.1 Mechanism

The coordination interaction between metal ions and hydrogel matrices introduces dynamic coordination bonds, significantly improving the mechanical integrity, structural resilience, and antibacterial performance of hydrogels, addressing the intrinsic weaknesses of conventional formulations.^147,148^ Various metal ions exhibit potent antibacterial activity through multiple mechanisms. For instance, Cu^2+^ interacts with sulfhydryl groups on bacterial proteins, disrupt bacterial membrane permeability, and induce the generation of reactive oxygen species (ROS). These processes collectively lead to protein oxidation, DNA degradation, and cell division arrest, ultimately triggering bacterial cell death. Similarly, Cu^2+^ can alter the structure and function of cell wall proteins, resulting in bacterial membrane rupture.^149^ In contrast, Ag^+^ binds to bacterial membranes, impair their structural integrity, and generate free radicals, thereby further exacerbating ROS-mediated bacterial damage.^113^ By integrating specific metal ions into hydrogel matrices, the hydrogel systems can exert photothermal therapy (PTT) effects, enabling localized thermal ablation of pathogens with negligible induction of bacterial drug resistance.^150,151^ For instance, the coordination of quercetin (Que) with Fe^3+^ (forming Fe-catechol complexes) has been proven to confer efficient photothermal conversion properties, thus enhancing antibacterial efficacy.^152^

#### 3.3.2 Examples

The GCP Hydrogel^153^

**Material Design:** The GCP hybrid hydrogel was fabricated by integrating GelMA with a copper-protocatechualdehyde (CuPA) coordination network. The mixture was subjected to UV irradiation in the presence of the photoinitiator lithium phenyl-2,4,6-trimethylbenzoylphosphinate (LAP) to induce photo-crosslinking and form a stable, uniform hydrogel matrix.

**Antibacterial Mechanism:** Cu^2+^ modifies bacterial cell membrane permeability by interacting with sulfhydryl groups and generates ROS.

**Performance:** The GCP hydrogel demonstrated strong antibacterial activity, reducing the survival rates of both *Staphylococcus aureus* and *Escherichia coli* *in vitro*. Compared to the control hydrogel of GC, the GCP system exhibited substantially improved antibacterial performance, highlighting the synergistic effect of the Cu^2+^-catechol coordination network.^153^

#### 3.3.3 Performance Comparison

Some metal-coordinated hydrogels antibacterial mechanisms and performances are summarized in Table S6.^152–165^

#### 3.3.4 Critical Appraisal

Antibacterial rates above 95–99% are frequently reported, but they are often measured against planktonic laboratory strains under irradiation conditions that are difficult to reproduce clinically.^152–165^ A useful study should report irradiance, exposure time, maximum tissue-facing temperature, clinically relevant biofilm or resistant isolates, and mammalian-cell viability at the bactericidal dose.^151–159^ Temperature-matched and ion-release-matched controls are essential to distinguish photothermal killing from metal-specific chemistry. Chronic-wound models should also assess re-epithelialization, scar quality, microbiome disturbance, and retained metal after the infection has resolved.

### 3.4 Comparative Value and Boundaries of the Platform

Across the three application areas, the most defensible advantage of a metal-coordinated hydrogel is spatial control: the network can retain a catalytic, ionic, or imaging function at a wound, joint, or resection cavity while maintaining conformal contact. A metal-free hydrogel may provide equal or better mechanical support when no metal-dependent mechanism is needed, whereas an unconfined metal nanoparticle may offer stronger immediate activity but poorer local retention. Current studies rarely test these alternatives head-to-head. The field should therefore shift from demonstrating multifunctionality to identifying the minimum metal exposure that adds a clinically meaningful benefit over an appropriate hydrogel-only or standard-of-care comparator. Applications beyond the therapeutic scope considered here may be promising, but they require separate critical evaluation. Quantitative performance comparisons across representative systems are summarized in Table S7.^13,32,77,120,121,132,143,154,159^

## 4 Translational Challenges and Design Criteria

### 4.1 Metal Pharmacokinetics, Degradation, and Long-Term Safety

A release study in buffer does not constitute a pharmacokinetic study. Both total and labile metal should be measured in the depot, blood, urine or feces, draining lymph nodes, and clearance organs, together with the intact complex and major ligand or degradation products when feasible. Essential elements are not intrinsically safe at local supraphysiological doses, and a degradable polymer does not ensure rapid or harmless metal clearance.^34,70,166^ Mass loss, swelling, molecular-weight change, and chemical identity of soluble fragments should be reported together, because each describes a different aspect of degradation.

### 4.2 Manufacturing, Sterilization, and Reproducibility

Metal-to-ligand ratio, order and rate of mixing, pH, ionic strength, oxygen exposure, polymer substitution, and precursor aging can markedly affect the product characteristics and speciation. Scale-up also changes mixing and heat transfer and may shift *in situ* nucleation. Sterilization cannot be treated as a final administrative step: filtration is not applicable for preformed gels, while steam, irradiation, and ethylene oxide may change coordination state, molecular weight, rheology, swelling, and release.^35,167^ Critical quality attributes should therefore include free and total metal, oxidation state, gelation time, viscoelasticity, extractables, endotoxin, sterility, release, and potency, with acceptance ranges defined before animal studies are undertaken.

### 4.3 Regulatory and Clinical Evaluation

The regulatory pathway will depend on whether the principal action is physical, pharmacological, or a combination of both, but the evidence needs to be product-specific in every case. Biological evaluation should be guided by a risk-management framework based on contact site and duration rather than a generic cytocompatibility assay. For metal-coordinated systems, the evaluation should be complemented by assessments of extractables and leachables, degradation products, local and systemic toxicokinetics, immunotoxicity where relevant, and repeated-dose or long-term implantation studies. Clinically meaningful comparators, administration and retrieval procedures, shelf-life, and usability should be addressed early. Short-residence wound dressings and local depots with measurable clearance are more plausible near-term options than permanently implanted, compositionally complex multifunctional systems.^5,35,70^

## 5 Conclusion and Outlook

Metal coordination is most useful when it performs a function that a polymer network cannot provide alone—catalysis, controlled ion delivery, optical or magnetic readout, radionuclide retention, or dynamic sacrificial crosslinking—and when the hydrogel measurably improves localization or handling of that function. The four integration strategies should therefore be viewed as design choices with different exposure and manufacturing consequences, rather than a hierarchy. Physical embedding favors simple, short-residence systems; covalent grafting favors retained molecular functions; coordination crosslinking favors dynamic mechanics and injectability; and *in situ* growth favors dispersed coordination phases when precursor control is feasible.

The most credible near-term opportunities are topical antibacterial dressings, superficial light-responsive materials, and localized depots in accessible cavities, where dose, exposure, and removal can be monitored. Joint and postoperative tumor applications are promising but require longer follow-up, realistic loading conditions, organ-level metal pharmacokinetics, and head-to-head comparison with standard care. Permanently implanted or repeatedly dosed systems would require a substantially stronger account of degradation products and cumulative metal burden.

Progress now depends less on adding functions than on testing the right causal and translational questions. Studies should identify the minimum effective metal exposure, report coordinated and labile fractions, use mechanism-matched controls, and link molecular coordination to network mechanics, release, and outcome. Formulations that retain an advantage after sterilization, scale-up, and clinically relevant comparison will offer a more convincing route from coordination chemistry to patient benefit.^5,34,70,166^

## Supplementary Material



## Notes

Table S1: chemically grafted systems and properties; Table S2: coordination crosslinking systems and properties; Table S3L: in situ synthesis systems and properties; Table S4: conditional comparison of the four integration strategies; Table S5: metal-coordinated hydrogels for osteoarthritis and osteochondral repair; Table S6: metal-coordinated hydrogels for antibacterial therapy; Table S7: quantitative performance and evidence gaps of representative systems (PDF)
