# Supplementary material for: Multifunctional Metal-Coordinated Hydrogels: Controlled Synthesis and Advances in Anti-Inflammatory, Anticancer, and Antibacterial Applications
Source: ACS Omega. 2026 Sep 8;11(37):54828–46. doi: 10.1021/acsomega.6c06631 (PMC13613481; doi:10.1021/acsomega.6c06631)
Supplement: Supplementary file 1 [file ao6c06631_si_001.pdf]

Multifunctional Metal-Coordinated Hydrogels: Controlled Synthesis and Advances in Anti-Inflammatory, Anticancer, and Antibacterial Applications

Junjia Gao[b,1], Zeng-En Jin[c,1], Yikuan Li[a,1], Chenxin Gu[d], Chunmei Zhi[a], Wei Wu[b], Qing Chen [a*], Sen Gao [a*]

[a] School of Pharmacy, Shenyang Medical College, Shenyang 110034, People's Republic of China;

[b] The Second Affiliated Hospital of Shenyang Medical College, Shenyang 110024, People's Republic of China;

[c] Central Hospital Affiliated to Shenyang Medical College, Shenyang 110024, People's Republic of China;

[d] Research Center for Analytical Sciences, Department of Chemistry, College of Sciences, Northeastern University, Shenyang 110819, People's Republic of China

[*] Corresponding authors.

E-mail: chenqing0906@symc.edu.cn (Q. Chen); gaosen@symc.edu.cn (S. Gao)

Tel: +86 24 62217511; Fax: +86 24 62215711

[1] These authors contributed equally to this work.

Table S1. Chemically Grafted Systems and Properties.

Representative System	Metal Ion	Ligand Type	Complex Type	Hydrogel Matrix	Fabrication Method	Key Functionality	Reference
GMS-PVA hydrogel	Fe^{3+}	Catechol	Fe^{3+}-catechol	PVA	Grafting, complexation and salting-out	High strength, toughness, conductivity	[78]
Ni-terpyridine PAAMa hydrogel	Ni^{2+}	Terpyridine	Ni^{2+}-terpyridine bis-complex	PAAMa (acrylic acid-maleic anhydride copolymer)	Grafting NH_2-Tpy onto PAAMa + coordination with $NiCl_2$	Thermo-responsive, reversible sol-gel transition	[79]
CEG-Fe^{3+} hydrogel	Fe^{3+}	Gallic acid (pyrogallol)	Fe^{3+}-pyrogallol (mono-/bis-/tris-complexes)	Carboxymethyl cellulose (CMC)	Grafting GA onto CMC-EDA + chelation with $FeCl_3$	Self-healing, tissue adhesion, antibacterial and antioxidant activity, and hemostasis	[80]
ODF-Met hydrogel	Fe^{3+}	Dopamine (catechol)	Fe^{3+}-catechol (mono-/bis-/tris-complexes)	Oxidized hyaluronic acid (OHA)	Grafting dopamine onto OHA + imine conjugation with metformin + Fe^{3+} coordination	pH-responsive drug release, photothermal antibacterial, self-healing, adhesive	[81]
ODMTI hydrogel	Fe^{3+}, Zn^{2+}, Mg^{2+}, Ca^{2+}	Dopamine (catechol)	Metal-catechol complexes	Oxidized dextran (ODex) + gelatin (DG)	Schiff base crosslinking + metal coordination	Low-swelling, super-adhesive, antioxidative, neuroregenerative	[82]

Table S2. Coordination Crosslinking Systems and Properties.

Representative System	Metal Ion	Ligand Type	Complex Type	Hydrogel Matrix	Fabrication Method	Key Functionality	Reference
P(AAm-co-AMPS) Hydrogel	Zr^{4+}	SO_3^- from AMPS monomers	Sulfonate-Zr^{4+} coordination bonds	P(AAm-co-AMPS)	Swollen hydrogel sequentially immersed in $ZrOCl_2$ solution and deionized water	High strength, dynamic responsiveness, and self-recovery	[13]
Zn^{2+}-FA/PDA Hydrogel	Zn^{2+}	Carboxyl groups from folic acid, catechol groups from PDA	Zn^{2+} coordination with FA/PDA	FA/PDA organic network	Zn^{2+} simultaneously chelates FA carboxyl and PDA catechol groups	Dual-light-triggered synergistic antibacterial action and enhanced wound healing	[32]
Fe^{3+}-P(AAm-co-AAc) Hydrogel	Fe^{3+}	-COOH from acrylic acid units	Carboxyl-Fe^{3+} coordination complexes	PAAM-acrylic acid copolymer, P(AAm-co-AAc)	Copolymer film immersed in $FeCl_3$ solution, forming networks *via* diffusive coordination bonding	High strength and dynamic responsiveness	[66]
Ni^{2+}-btp-Star PEG Hydrogel	Ni^{2+}	btp	Binuclear complexes	Tetra-arm star polyethylene glycol	Crosslinking *via* dynamic Ni^{2+}-btp coordination bonds	Rapid self-healing and high strain resistance	[67]

Table S3. *In Situ* Synthesis Systems and Properties.

Representative System	Metal Ion	Ligand Type	Complex Type	Hydrogel Matrix	Fabrication Method	Key Functionality	Reference
Eu^{3+}/Tb^{3+} complexes/PAAm Hydrogels	Eu^{3+} and Tb^{3+}	2,6-Pyridinedicarboxylic acid	Lanthanide metal complex	PAAm	Complexes added to the monomer solution pre-polymerization, copolymerized with AAM to form a hydrogel network	Synergistic high adhesion energy and stable luminescence	[83]
HKUST-1-alginate	Cu^{2+}	Trimesic acid	HKUST-1	Sodium alginate	In-situ growth in ligand solution	Shapeable dye adsorbent	[88]
HKUST-1/SA-CMC-PEI Hydrogel	Cu^{2+}	H_3BTC	Copper-based MOF	SA, CMC, PEI	Ligand diffusion triggers *in situ* MOF crystallization within the network	Mechanical toughness combined with smart antifouling and anticorrosion properties	[89]
ZIF-8@TOCNF Hydrogel, MIL-100(Fe)@TOCNF Hydrogel	Zn^{2+}, Fe^{2+}	Hmim, H_3BTC	ZIF-8, MIL-100(Fe)	TOCNF	Zn^{2+}/Fe^{2+} coordinate with -COOH on TOCNF, MOFs grow on fiber surfaces	Shear-thinning behavior and pH-responsive drug release	[90]
Au(I)/Ag(I) Hydrogels	Au^{+}, Ag^{+}	-SH from thiolated four-arm PEG	Metal(I)-thiolate complexes	PEG	*In situ* coordination and oxidative crosslinking	Self-healing and frequency-dependent mechanical properties	[91]
Lanthanide MOF gels	Eu^{3+}, Tb^{3+}, Dy^{3+}	5-boronoisophthalic acid	Ln-MOFs	Self-supporting MOF network	Solvothermal assembly of Ln^{3+} and ligand	Intrinsic tunable emission and detection	[95]
ZIF-8/chitosan composites	Zn^{2+}	2-methylimidazole	ZIF-8	Chitosan	*In-situ* growth in chitosan hydrogel	Enhanced CO_2 adsorption	[83]

Table S4. Conditional Comparison of the Four Integration Strategies.

Strategy	Defining event	Distribution / retention	Release behavior	Mechanical contribution	Responsiveness	Scalability / reproducibility	Best-suited use	Main failure mode
Physical Embedding	Preformed complex or particle retained without a covalent polymer bond	Simple but sedimentation and leakage depend on size and affinity	Often diffusion- or erosion-dominated; burst release possible	May be neutral or reinforcing; particle loading can also embrittle	Rapid response feasible when cargo remains accessible	Usually the most scalable; mixing history and particle dispersion remain critical	Short-residence depot, dressing, sensor, proof of concept	Aggregation, burst release, or poor dose uniformity
Chemical Grafting	Complex or ligand covalently coupled to the polymer	High retention of intact crosslinker when conversion is complete	Low until linker or complex is cleaved; fragments may then release rapidly	Defined crosslinks can stiffen the network; permanent bonds may reduce stress relaxation	Triggered cleavage can be precise	More synthetic and purification steps; conversion and residuals require control	Retained optical or mechanical motif, on-demand cleavage	Incomplete coupling, altered complex activity, toxic cleavage products
Coordination Crosslinking	Metal–ligand bonds form network junctions	Molecular distribution possible; stability sensitive to biological competitors	Coupled to bond exchange, dilution, pH, redox state, and erosion	Dynamic bonds can provide toughness, self-healing, and injectability	High when bond lifetime changes with the trigger	Aqueous processing is attractive; stoichiometry, pH, and oxidation state are critical	Dynamic tissue interface, injectable depot, wound dressing	Creep, decomplexation, or uncontrolled metal exchange
In situ Synthesis	Coordination particle or complex nucleates inside a polymer network	Can reduce aggregation and improve interfacial contact	Coupled particle dissolution and matrix transport	May reinforce or disrupt the matrix depending on nucleation and loading	Framework dissolution can respond to pH or ligands	One-pot processing can be mild, but scale changes mixing and nucleation	MOF–hydrogel composite or spatially patterned coordination phase	Gradients, residual precursor, variable crystal size or conversion

Table S5. Metal-Coordinated Hydrogels for Osteoarthritis and Osteochondral Repair.

Material system	Metal category	Anti-inflammatory mechanism	Performance	Reference
CuTA@SF hydrogel	Cu (Copper)	Nrf2 pathway activation, upregulating SOD-1/HO-1, scavenging ROS, and inhibiting IL-1β-induced ECM degradation	Promotes BMSC osteogenesis, enhances GAG deposition, improves BMD/BV/TV, and achieves highest histological repair scores in rabbit OCD model	[77]
PBT-Gel	Ga^{3+}-carboxylate complex	1. Activation of TGF-β/Smad signaling; 2. antioxidant and ROS-scavenging activity	1. Restoration of cartilage matrix 2. Maintenance of ECM homeostasis 3. Reduction in inflammatory response	[118]
^{177}Lu/AMP@CG hydrogel	^{177}Lu-AMP complex	1. Induce inflammatory cell apoptosis 2. Clear ROS	1. Significant reduction in inflammatory cytokines and ROS levels. 2. Indicators such as bone volume fraction and bone surface area density are normalized	[120]
Fe^{3+}-GSH-HA Gel	Fe^{3+}-GSH complex	Inhibit MMP-13	Significant inhibition of MMP-13 activity, surpasses commercial products	[121]

Table S6. Metal-Coordinated Hydrogels for Antibacterial Therapy.

Material system	Metal category	Antibacterial mechanism	Performance	Reference
Que-Fe^{3+}-BNN6-CMCS-OHA Gel	Fe^{3+}	PTT	High bactericidal rates against *E. coli*, *S. aureus,* and MRSA	[152]
GCP Gel	CuPA complex	Disrupt bacterial membrane permeability by Cu^{2+}	Significant reduction in the survival rate of *S. aureus* and *E. coli* in comparison to the control hydrogel	[153]
Fe^{3+}-Mn^{2+}-LDH-panis-QCS-TA Gel	1. Fe^{3+}<br>2. Mn^{2+}	1. CDT<br>2. PTT	Over 95% clearance of *S. aureus* and *E. coli,* with near-complete eradication of MRSA	[154]
CO_2-PDA-Fe^{3+}-CBSP Gel	Fe^{3+}	Disrupt bacterial cellular structures	Demonstration of over 90% antibacterial efficacy against *S. aureus* and *E. coli*	[155]
Rhein-Cu^{2+}/Zn^{2+}-EW-ODex-Gel	1. Cu^{2+}<br>2. Zn^{2+}	1. Disrupt bacterial membrane by Cu^{2+}<br>2. Damages bacterial cell walls by Zn^{2+}	High antibacterial performance of the hydrogel against *E. coli* and MRSA	[156]
Mn-terpy-COOH/OH-PG Gel	Mn(I) Carbonyl complex	Inhibits bacterial growth by CO	Light-responsive antibacterial activity, exhibiting significant efficacy under illumination and no antibacterial effect in the absence of light	[157]
THBA-Fe-CMCS Gel	THBA@Fe	PTT	98% and 99% elimination of *S. aureus* and *E. coli*, respectively, with complete infection clearance within 3 days	[159]
CHI(C)-Fe^{3+}-P(NIPAM-AM) Gel	Fe^{3+}-catechol complex	CDT	Survival rates of *E. coli* and *S. aureus* approach 0%, significantly outperforming the control group	[158]
AgCl-NPs-poly (SBMA-HEA) Gel	Ag^{+}	Antibacterial effects by Ag^{+}	Antibacterial activity of the 0.20NPs-PSH hydrogel against *S. aureus, E. coli*, *Pseudomonas aeruginosa* (P. aeruginosa), and MRSA, with a longer onset time compared to commercial dressings	[160]
Cu/OBC hydrogels	Cu^{2+}	Cu^{2+} effects and GSH-activated Fenton-like • OH	Broad-spectrum antibacterial and antifungal activity	[162]
Cu^{+}/Cu^{2+}-HA-NCSN Gel	Cu^{+}/Cu^{2+} nanoclusters	PTT	Bactericidal efficacy against *S. aureus* and *E. coli*, demonstrating performance superior to the control group	[161]
AgNPs-Zn^{2+}-CEC-OSA Gel	Silver nanoparticles and Zn^{2+}	1. Disrupts bacterial cell membranes by Ag^{+}<br>2. Induce the generation of ROS by Zn^{2+}	Inhibition of *E. coli* and *S. aureus* growth, with antibacterial efficacy 2.5 times higher than that of conventional hydrogels	[163]
EGCG-Cu-Lap-Gelatin Gel	Cu-EGCG complex	Disrupt bacterial biofilms and induce bacterial apoptosis by Cu^{2+}	Reduction of colony counts of *Porphyromonas gingivalis* and *S. aureus*, demonstrating higher antimicrobial efficacy compared to the	[165]

Material system	Metal category	Antibacterial mechanism	Performance	Reference
			blank hydrogel	
Zn-GA-Supramol hydrogel	Zn-GA	Inhibit bacterial proliferation by Zn^{2+}	Significantly lower minimum inhibitory concentration compared to GA monomer and Zn^{2+}	[164]

Table S7. Quantitative Performance and Evidence Gaps of Representative Systems.

Systems	Application	Strategy	Metal loading / distribution	Gelation / mechanics	Material or release metric	Release / retention	In vitro endpoint	In vivo endpoint	Model / follow-up	Primary gap	Ref.
CuTA@SF hydrogel	Osteochondral regeneration	Physical embedding	0.21% Cu (XPS); uniform (EDS)	HRP/H_2O_2 crosslinked; G′ > G″; stable modulus	31.89% TA release (3 d); 0.96 mg TA (6 d)	Sustained release (6d)	Antioxidant; antibacterial; pro-osteogenic; anti-inflammatory	Enhanced bone & cartilage regeneration (12 w)	Rabbit femoral condyle; 12 weeks	Long-term mechanical stability in vivo; direct comparison with clinical products	[77]
EB@CuHsp hydrogel	Wound healing, antibacterial, pH monitoring	Physical embedding	Cu-MOF ~823 nm, uniform dispersion	UV photocrosslinking; G′ > G″; compressive strength ~30×	Cu^{2+}/Hsp/BA cumulative release	Cu/Hsp >60% in 1 d; BA within 4 h	>99% MRSA kill; hemolysis <5%; HUVEC >95% viability	~99% wound closure at 14 d; re-epithelialization	Mouse MRSA-infected full-thickness wound; 14 d	L929 cytotoxicity at high Cu; long-term biosafety unclear	[20]
RuMePhen gelatin hydrogel	Antibacterial photopolymerized dressing	Physical embedding	Ru complex 0.1–0.5 mM in matrix	Visible light (400 klux, 3 min); G′ > ~60 Pa; self-healing granular	Leaching not quantified	NR	Antibacterial at ≥0.3 mM; cytocompatible ≤0.5 mM (HDFs)	Injection into porcine skin (ex vivo only)	Porcine skin cavity; no in vivo healing follow-up	No in vivo wound model; MBC higher than free RuMePhen	[21]
EGCG@FSG hydrogel (Gel@NPs)	Diabetic foot ulcer (anti-inflammatory, antibacterial, angiogenic)	Physical embedding	Fe^{3+} NPs ~233 nm; loaded with SAB/GOx	Schiff base (QCS/OFu); G′ > G″; injectable; self-healing	SAB and GOx pH-responsive release	>60% at 72 h (pH 5.5)	ROS scavenging 80-92%; M2 polarization; HUVEC tube formation	Complete closure at 14 d; reduced inflammation; angiogenesis	Rat DFU (STZ + S. aureus); 14 d	Multi-step synthesis; only small animal model tested	[33]
CEG-Fe^{3+} Hydrogel	Wound dressing	Chemical Grafting	Fe^{3+} coordinated to grafted GA (pH-dependent mono/bi/tri) / Homogeneous in network	Compressive stress 1.5 MPa, strain 1720% (pH 9); G′~10^4 Pa at 1% strain	Swelling ~240% (pH 3) to ~450% (pH 9) at 48h; Gel fraction 90.9% (pH 3) to 78.6% (pH 9)	NR (no drug loaded in this system)	DPPH scavenging 58.3% at 0.02% conc.; Antibacterial zone inhibition; Cell viability 84%	Adhesion 11.44 kPa on pigskin; Tail hemostasis; Hemolysis <5%	Rat tail amputation / acute (follow-up time NR)	No active therapeutic agent loaded; only acute hemostasis model evaluated	[80]
ODF-Met Hydrogel	Diabetic infected wound healing	Chemical Grafting	Fe^{3+} coordinated to grafted dopamine	G′~10^3 Pa at 1% strain; Adhesion 28.6-30.3 kPa on	Metformin loading: 0.5, 1.0, 2.0 mM;	pH-responsive release: 87.9% in 3h	Cell viability ~107% (ODF-1Met); Intracellular ROS	Wound closure 100% at day 14; HE/Masson	STZ-induced diabetic rat (FBG>17 mM) / 21	Complex multi-step synthesis; long-term biosafety and	[81]

Systems	Application	Strategy	Metal loading / distribution	Gelation / mechanics	Material or release metric	Release / retention	In vitro endpoint	In vivo endpoint	Model / follow-up	Primary gap	Ref.
			(DA:Fe^{3+}=3:1 optimal) / Homogeneous in network	pigskin	Swelling ~126-130% at 12h; Degradation complete by day 14 (pH 7.4)	(pH 5.5); 85.0% in 60h (pH 7.4)	scavenging; M2 macrophage polarization; Pro-angiogenic gene upregulation	histology; IHC (CD86/CD206, CD31, VEGF, TGF-β3)	days	degradation products not fully addressed	
P(AAm-co-AMPS) hydrogel	Tubular grasper / soft connector	Coordination crosslinking	Zr^{4+} (0.05-1 M); S/Zr ratio ~0.5	Tunable E (0.4-28.5 MPa), σb (2.3-11.6 MPa), εb (215-995%)	Mechanical properties (E, σb, εb, G)	NR (not a drug delivery system)	Low cytotoxicity	Vascular anastomosis in rabbit	Rabbit model; follow-up NR	Long-term *in vivo* degradation/biocompatibility	[13]
DFT-C/ZnO injectable hydrogel	Wound healing & antibacterial dressing	Coordination crosslinking	Zn^{2+} coordinated; release over 12 d	Rapid gelation; G′ > G″ stable moduli	Zn^{2+} release, ROS & photothermal heat	Cumulative Zn^{2+} release for 12 d	Antibacterial 99.999%; cell viability >80 %; gene upregulation	Wound closure 95.7% at 10 d; improved histology	Rat infected wound model; 10-day follow-up	Long-term degradation and biosafety unclear	[32]
^{177}Lu/AMP@CG Hydrogel	Anti-inflammatory & Chondroprotective (Osteoarthritis)	Coordination crosslinking + Physical embedding	NR / 0.68% Lu in hydrogel	Thermosensitive; LCGT ~37°C; G′ > G″ at 37°C	Cumulative ^{177}Lu release < 30% at 16 days	In joint: 86% retained at 16 days post-injection	Reduced IL-1β, TNF-α, ROS; Increased IL-10, GAG, Col II	Reduced synovitis; Cartilage protection; Reduced osteophytes	ACLT/MMx rat model / 6 weeks	Lack of long-term safety & large animal validation	[120]
Fe^{3+}-GSH crosslinked HA hydrogel	Osteoarthritis therapy	Coordination crosslinking	NR (Low Fe content)	Gelation completed within 1 minute; Storage modulus of ~120 Pa; Self-healing behaviour; Withstands shear strain up to 300 %	NR (Drug-free system)	NR	Tolerated by ATDC5 chondrocytes; Reduced MMP-13 activity to 92.9 % of control after 24 hours	Reduced total MMP activity in osteoarthritic synovial fluid to 81.0 % of control after 24 hours	In vitro and ex vivo / 24 hours	Lack of in vivo efficacy and biodegradation data	[121]
N-Pep-Mn gel (+αPD-1)	Antitumor immunotherapy	Coordination crosslinking	Mn 5.3 at.% / EDS uniform	G′ > G″, shear-thinning, injectable	Porous network, KD 4.08 μM	Mn 40% at 138h (pH 5.5); αPD-1 168h	DC maturation, M1 polarization, STING activation	Tumor suppression, CD8+ T infil., memory	H22 tumor BALB/c / 35-60 d	Long-term toxicity & clinical translation	[132]

Systems	Application	Strategy	Metal loading / distribution	Gelation / mechanics	Material or release metric	Release / retention	In vitro endpoint	In vivo endpoint	Model / follow-up	Primary gap	Ref.
Cel hydrogel	Anticancer (chemo-immunotherapy)	Coordination crosslinking	Cu ~10 µg/g in tumor; low in organs	G′ > G″, shear-thinning, self-healing, injectable	Particle size ~218 nm; zeta potential -37.6 mV	Sustained release from gel degradation	Apoptosis (35.8%), ROS increase, GSH decrease, Bax/Bcl-2	Tumor inhibition 75-91%; T cell infiltration increase; IFN-γ increase; combined with aPD-L1	BALB/c mice, 4T1 unilateral/ bilateral; 14 d	Local injection only; long-term safety and translation needed	[143]
QCS-TA/LDH-panis	Anti-biofilm wound healing & real-time monitoring	Coordination crosslinking	Fe/Mn in LDH-panis, uniformly distributed	Gelation time 34-200 s; G′ 239-627 Pa	NIR photothermal conversion efficiency 64.3%; •OH generation	•OH release under acidic pH/H_2O_2	>95% biofilm eradication (S. aureus/E. coli)	98.2% wound closure in 12 days	Mouse dorsal infected wound model; 12 days	Long-term biodegradation & clinical translation need evaluation	[154]
CTF hydrogel	Infected wound healing	Coordination crosslinking	Fe^{3+}, 0.05-0.15 M THBA @Fe	G′ > G″, rapid gelation (34-81 min)	NR	NR (No drug release)	99% bacterial kill rate (E. coli)	91.4% wound healing rate	S. aureus infected mouse, 14 days	Long-term stability and degradation	[159]
Nacre-inspired HMC	Antifouling & anti-MIC	Coordination crosslinking & *In situ* synthesis	Cu uniformly distributed in ~70 µm interpenetrating network	Multi-scale crosslinking enhances strength & toughness	Cu^{2+} release (ICP-MS)	Sustained ~27.5 µg cm^{-2} d^{-1}; pH-responsive burst at pH 4	Anti-protein, antibacterial (>99% for several strains), anti-algae	NR	Seawater / P. aeruginosa, etc. / up to 14 days	Lack of long-term in vivo efficacy & environmental fate studies	[88]
CelloZIF8 3D Scaffold	Drug delivery (curcumin)	*In situ* synthesis & Coordination	Zn homogeneously distributed in TOCNF scaffold	Shear-thinning; G′ up to 4.8×10^5 Pa with increasing ZIF-8	Curcumin release (UV-vis); BET surface area up to 900 m^2 g^{-1}	pH-controlled release: >90% within 5 h at pH 5.5; negligible at pH 7.4/10	Drug release kinetics; cell viability (fibroblasts)	NR	In vitro / pH 5.5, 7.4, 10 / 30 h release	Lack of in vivo efficacy, biodegradation & tissue integration studies	[89]
Au/Ag-thiolate Hydrogel	Self-healing injectable biomaterial	Coordination crosslinking	Au or Ag ions; loading controlled by thiol:metal ratio	Injectable; frequency-dependent stiffness; G′ tunable from ~500	Rheological recovery (G′ & G″); compression	Self-healing within 1 min; ~90% mechanical	Fibroblast proliferation (slower but viable); Live/Dead & Ki67 staining	NR	In vitro / Human dermal fibroblasts / 7 days	Lack of in vivo biocompatibility, bioactivity (e.g., RGD grafting)	[90]

Systems	Application	Strategy	Metal loading / distribution	Gelation / mechanics	Material or release metric	Release / retention	In vitro endpoint	In vivo endpoint	Model / follow-up	Primary gap	Ref.
			(5–60 mol%)	to >4000 Pa	test	recovery after damage				& long-term stability	

NR, not reported in a form permitting direct comparison.
